# Application of Just-in-Time Adaptive Interventions in Dietary Health Management: Systematic Review

**DOI:** 10.2196/92139

**Published:** 2026-07-16

**Authors:** Chang Ying Li, Zhen Zhu Jiao, Li Qin Zhang, Hang Li, Meng Yao Wang, Wen Hui Guo, Yu Xin Wang, Yang Wang

**Affiliations:** 1 School of Nursing, Changchun University of Chinese Medicine, No 1035 Boshuo Road, Changchun Jingyue National High - Tech Industrial Development Zone, Changchun, Jilin, 130117, China, 86 15943030083; 2 Encephalopathy Center, The Affiliated Hospital to Changchun University of Chinese Medicine, Changchun, Jilin, China

**Keywords:** immediate adaptive intervention, adaptation intervention, dietary management, dietary health, ecological momentary intervention, digital technology

## Abstract

**Background:**

Just-in-time adaptive interventions (JITAIs) use real-time data to deliver personalized support at moments of heightened need and may improve dietary behaviors in real-world settings.

**Objective:**

The aim of this study is to systematically review the application, characteristics, and effectiveness of JITAIs in dietary health management.

**Methods:**

We included human studies evaluating JITAIs-based dietary interventions delivered through digital platforms that used real-time or near–real-time data to tailor intervention content, timing, or intensity. Eligible studies reported at least one behavioral, engagement, physiological, or clinical outcome; reviews, protocols, editorials, commentaries, and studies without outcome data were excluded. We searched PubMed, Embase, Scopus, CINAHL, Web of Science, ClinicalTrials.gov, WHO ICTRP (International Clinical Trials Registry Platform), and ISRCTN (International Standard Randomized Controlled Trial Number) from inception. The initial search was conducted on August 20, 2025, and updated on March 16, 2026; reference lists were also screened manually. Two reviewers independently screened studies and extracted data. Methodological quality was assessed using the 2018 Mixed Methods Appraisal Tool, and reporting quality was assessed using the Mobile Health Evidence Reporting and Assessment checklist. Because of substantial heterogeneity, findings were synthesized narratively. The review was registered in PROSPERO (International Prospective Register of Systematic Reviews; CRD420261285292).

**Results:**

Twenty studies involving 2948 participants were included. Target populations comprised individuals with overweight or obesity, chronic conditions, and eating disorders and the general population engaged in dietary management. Interventions were mainly delivered via smartphone apps, SMS text messaging, wearable-device feedback, and context-triggered notifications. More consistent benefits were observed for proximal behavioral and process outcomes, including fruit and vegetable intake, sodium-restriction behaviors, drinking automaticity, self-monitoring, eating-related behaviors, and responsiveness to prompts. Some studies also reported improvements in distal clinical outcomes, such as body weight, BMI, waist circumference, blood pressure, blood glucose, and selected biochemical indicators. However, these findings were inconsistent, and most studies did not show clear between-group advantages. Common implementation barriers included device incompatibility, variability in digital literacy, geolocation or signal limitations, and burden from frequent prompts.

**Conclusions:**

JITAIs-based dietary interventions appear promising for supporting timely and individualized dietary behavior change, particularly for proximal behavioral outcomes, although evidence for sustained clinical effects remains inconsistent. This review contributes to the JITAIs literature by examining dietary health management as a distinct application domain and by synthesizing evidence that has otherwise been dispersed across broader reviews of digital behavior change and weight management. By integrating intervention characteristics, delivery approaches, triggering mechanisms, and effects across diverse populations, it clarifies methodological and implementation gaps and informs more standardized intervention design and reporting. These findings support the development of scalable, context-sensitive digital dietary interventions for clinical care, chronic disease self-management, weight management, and public health nutrition.

## Introduction

Dietary health has become a major global public health challenge [1,2]. In recent years, accelerated urbanization and rapid lifestyle changes have contributed to the increasing prevalence of unhealthy dietary behaviors, such as high salt intake and insufficient fruit and vegetable consumption, thereby driving the rising incidence of chronic diseases including obesity, cardiovascular disease, and diabetes [3,4]. Dietary health management has been recognized as a key modifiable factor in many chronic conditions and can serve as an effective lifestyle strategy for both the prevention and management of chronic diseases [5,6]. In 2013, the World Health Organization (WHO) identified a 30% relative reduction in mean population salt/sodium intake by 2025 as one of the 9 voluntary global targets for the prevention and control of noncommunicable diseases [7]. However, as of 2022, this target had not yet been achieved globally. No country had met the proposed reduction goal, and only 9 countries, including Brazil, Chile, Spain, and Malaysia, had implemented comprehensive mandatory sodium reduction policies, while most countries continued to rely primarily on voluntary measures. Overall, global progress remained substantially behind the target requirements [8]. This situation suggests that existing dietary health intervention models remain insufficient to support long-term and sustained dietary behavior change. Although traditional face-to-face dietary interventions have shown some effectiveness in improving short-term dietary behaviors, their impact is constrained by limited adherence, low intervention frequency, and high resource demands, making them inadequate for meeting the need for continuous and personalized dietary health management [9,10]. Therefore, the development of scalable, personalized, and sustainable dietary health management strategies is of considerable public health importance for advancing global salt reduction targets and improving chronic disease prevention and control, while also offering a clear direction for optimizing future dietary health intervention models.

Just-in-time adaptive interventions (JITAIs) are an innovative digital behavior change intervention design that uses real-time user data to deliver personalized support when individuals are in states of heightened vulnerability or susceptibility, thereby maximizing intervention effectiveness [11,12]. This concept was proposed by Nahum-Shani et al [11,12], who also developed a systematic theoretical framework defining six core design elements of JITAIs: (1) proximal outcomes, referring to short-term and immediately observable behavioral or psychological changes; (2) distal outcomes, referring to long-term health outcomes, such as weight control or reduced chronic disease risk; (3) decision points, namely the specific moments at which the system determines whether an intervention should be delivered; (4) decision rules, which use prespecified logic, such as if–then rules or algorithmic models, to determine the type and content of the intervention; (5) intervention options, referring to the set of available intervention strategies; and (6) tailoring variables, which are the data sources used to personalize intervention decisions, including individuals’ internal states, such as emotions and motivation, as well as external contextual factors such as time, location, and environmental characteristics. Through the interaction of these elements, JITAIs are able to achieve a high degree of context sensitivity and personalized responsiveness in dynamically changing real-world settings, thereby distinguishing themselves from traditional digital interventions characterized by fixed timing and standardized content.

JITAIs leverage smartphones and wearable devices to continuously monitor individuals’ physiological indicators, behavioral patterns, and environmental context and provide intervention support matched to the current situation at the moments when support is most needed and most likely to facilitate behavior change [11,13]. Existing studies suggest that JITAIs are both feasible and potentially effective in promoting health behavior change. Unlike traditional fixed-schedule interventions, their core design features include real-time identification of intervention needs, dynamic adjustment of intervention content and timing, and system-triggered delivery of support [14-16]. To date, several reviews have focused on areas such as weight management [17], physical activity [18], and substance use [19], summarizing the application forms and preliminary effects of JITAIs in behavior change while also highlighting persistent challenges, including inconsistent terminology, incomplete reporting, and substantial variation in intervention design, all of which limit comparability and synthesis across studies [20,21]. Although dietary behavior is a key determinant of the development and progression of chronic diseases, systematic synthesis of the application of JITAIs in dietary health management remains limited. Considerable heterogeneity exists across studies in terms of intervention goals, technological implementation, and outcome measures, and the evidence base remains fragmented. In addition, some studies are constrained by limited automation and insufficient reporting, making it difficult to provide clear guidance for future research and practice [22]. Given the scarcity and heterogeneity of JITAIs studies in dietary management, as well as the inherent complexity of JITAIs design, the present systematic review seeks to synthesize the existing evidence, identify research gaps, and advance the development of this field. Specifically, this review aims to systematically examine the current application of JITAIs in dietary health management, with a particular focus on their intervention characteristics, implementation approaches, and effects across different outcome measures, in order to inform the optimization of JITAIs design and improve intervention effectiveness.

The primary research questions of this systematic review were as follows: (1) Which populations have been targeted? (2) What are the characteristics of JITAIs designed to promote dietary health management, including intervention duration, intervention content, delivery modality, and triggering mechanisms? (3) What outcomes have been used to evaluate the effectiveness of current JITAIs for dietary health management, and what effects have been observed on user-related outcomes?

## Methods

### Protocol and Registration

This systematic review was conducted and reported in accordance with the PRISMA (Preferred Reporting Items for Systematic Reviews and Meta-Analyses) 2020 statement (Checklist 1) [23]. Given that no meta-analysis was performed, the SWiM (Synthesis Without Meta-Analysis) reporting guideline was also applied [24]. EndNote X21 (Clarivate Analytics) was used for literature management and screening, and the study selection process followed the PRISMA-S (Preferred Reporting Items for Systematic Reviews and Meta-Analyses—Literature Search Extension) guideline (Checklist 2) [25]. This systematic review has been registered with PROSPERO (International Prospective Register of Systematic Reviews; CRD420261285292).

### Eligibility Criteria

The inclusion and exclusion criteria were established according to the PICOS (population, intervention, comparator, outcomes, and study design) framework. In this review, dietary health management was operationally defined as interventions in which diet-related behavior change, dietary self-management, nutritional improvement, or health goals closely linked to dietary regulation constituted the primary intervention target. The inclusion criteria were as follows.

- Population: Individuals with diet-related health needs were included, including but not limited to those requiring dietary behavior change, chronic disease dietary management, or nutritional improvement.
- Intervention: JITAIs-based dietary interventions that used digital platforms, such as smartphones or wearable devices, to collect real-time individual data and dynamically adjust intervention content, timing, and intensity.
- Comparator: usual care, standard health education, traditional health education, digital interventions without JITAIs functions, or blank controls.
- Outcomes: Eligible studies were required to report at least one of the following categories of outcomes: (1) user engagement, such as adherence, frequency of use, or response rate, and/or (2) intervention effects, such as dietary behavior change or physiological and clinical outcomes.
- Study design: Original studies published in peer-reviewed journals were included, including randomized controlled trials (RCTs), nonrandomized controlled studies, feasibility studies, and pilot studies, using quantitative or mixed-methods designs.

The following studies were excluded: (1) studies not validated in human participants; (2) studies that did not report outcomes; (3) studies whose results did not include specific indicators explicitly describing the effects of JITAIs; and (4) publication types such as systematic reviews, editorials, commentaries, reviews, study protocols, and studies for which the full text was unavailable.

### Information Sources

Literature searches were conducted on March 16, 2026, across the following electronic databases: PubMed (NCBI), Embase (Elsevier), Scopus (Elsevier), CINAHL (EBSCOhost), and Web of Science Core Collection (Clarivate). Concurrently, the following clinical trial registries were searched on the same date: ClinicalTrials.gov, WHO ICTRP (International Clinical Trials Registry Platform), and ISRCTN (International Standard Randomized Controlled Trial Number). All databases were searched independently; no cross-database platform was used for simultaneous searching. Supplementary searches were performed by manually screening the reference lists of all included studies and relevant systematic reviews to identify potentially eligible missed studies. No additional or unpublished studies were sought by contacting authors, experts, manufacturers, or other institutions. No other targeted online or gray literature searches were conducted beyond registry searching and backward citation tracing.

### Search Strategy

The search strategy was developed and iteratively refined by CYL and ZZJ to optimize the search sensitivity for terms related to JITAIs and dietary health. The complete search strings for each database and registry, along with the final search date, are reported in Multimedia Appendix 1. The number of records identified from each information source is detailed in Multimedia Appendix 2. During the search phase, no restrictions were imposed a priori on language, publication date, document type, or study design to identify literature relevant to the research question as comprehensively as possible. Decisions regarding inclusion and exclusion were made primarily during title/abstract screening and full-text screening according to prespecified eligibility criteria. No published search filters were used or adapted in this study; search terms were independently developed and iteratively revised by the research team based on core concepts, including dietary health, dietary behavior, JITAIs, and ecological momentary intervention (EMI). The search strategy was not substantially derived from or reused from those of previous reviews. The search was initially run on August 20, 2025, and subsequently rerun across all databases and registries on March 16, 2026, to ensure up-to-date results. No external peer review of the search strategy was conducted prior to formal searches.

### Selection Process

All records from databases, registration platforms, and supplementary citation retrieval were imported into EndNote X21 for management. Duplicate references were first automatically identified by EndNote and then manually verified as needed based on bibliographic information, including title, authors, journal, year, and DOI. CYL was responsible for database searching and initial deduplication. Two reviewers independently screened titles and abstracts according to prespecified inclusion and exclusion criteria; any disagreements were resolved through discussion. During the main search phase, CYL conducted full-text eligibility assessment; uncertainties were independently reviewed by ZZJ. During the supplementary search phase, CYL and ZZJ independently screened full-text records obtained via citation retrieval; any disagreements were also resolved through discussion.

### Data Collection Process

The following information was extracted from each included study: (1) first author, publication year, and country; (2) study design; (3) sample size, population characteristics, and intervention setting; (4) recruitment methods; (5) participant characteristics, including age, sex, race/ethnicity, educational level, and socioeconomic status; (6) characteristics of JITAIs, including delivery modality, intervention duration, and triggering mechanism; and (7) outcome measures related to effectiveness.

### Study Risk of Bias Assessment

The methodological quality of the included studies was independently assessed using the 2018 version of the Mixed Methods Appraisal Tool (MMAT) [26]. This tool provides design-specific criteria for different study types, including RCTs and nonrandomized quantitative studies, with each item rated as “Yes,” “No,” or “Cannot tell.” Given that the MMAT emphasizes item-level appraisal rather than the calculation of an overall score, the methodological quality of each study was interpreted cautiously on the basis of its performance across individual items. The detailed assessment results are presented in Multimedia Appendix 3.

In addition, the Mobile Health Evidence Reporting and Assessment (mERA) checklist was used to further evaluate the reporting quality of the included studies [27]. This checklist covers domains such as intervention content, implementation context, and technical characteristics. Each item was assessed as “fully reported,” “partially reported,” or “not reported” to provide a more detailed reflection of the completeness of reporting for mobile health interventions. The quality assessment was conducted independently by CYL and ZZJ. Any disagreements were resolved through discussion, and if consensus could not be reached, a third reviewer YW made the final decision. The formal grading of the certainty of evidence for each outcome, such as with the GRADE (Grading of Recommendations Assessment, Development and Evaluation) approach, was not undertaken because of substantial heterogeneity across the included studies in terms of study design, intervention objectives, target populations, and outcomes, as well as the absence of statistical pooling at the outcome level. Instead, the strength of the evidence was interpreted narratively and cautiously, taking into account study design, methodological quality, reporting completeness, consistency, and directness.

### Synthesis Methods

Given the substantial heterogeneity across the included studies in terms of target populations, intervention formats, study designs, outcome measures, and follow-up duration, a meta-analysis was not conducted. Instead, a narrative synthesis was performed. Data synthesis was organized around 2 research questions: characteristics and intervention effectiveness of JITAI. To synthesize JITAI characteristics, studies were categorized according to delivery modality, triggering mechanism, active/passive data collection approach, and the name of the JITAI system. To synthesize intervention effectiveness, studies were grouped by target population and outcome type, and the direction and consistency of findings were compared. Outcomes with similar concepts but different wordings were harmonized into common categories. For studies with incompletely reported summary statistics, no numerical imputation or effect size conversion was undertaken; instead, findings were extracted narratively based on the original reports. The results were primarily presented through structured tables and narrative summaries. Because no statistical pooling was performed, no pooled effect estimates, heterogeneity statistics, or sensitivity analyses were calculated. Potential sources of heterogeneity were explored descriptively by comparing study design, target population, intervention characteristics, and outcome type. No substantive modifications were made to the prespecified synthesis framework during the review process. All studies meeting the inclusion criteria were included in the narrative synthesis. When findings were inconsistent across studies, greater emphasis was placed on study design, sample size, methodological quality, and direct relevance to the research questions. As no meta-analysis was performed and the synthesis was primarily narrative, no formal statistical assessment of reporting bias due to missing results was conducted, and the certainty of evidence was not formally graded using tools such as GRADE.

## Results

### Study Selection

**Figure 1.**
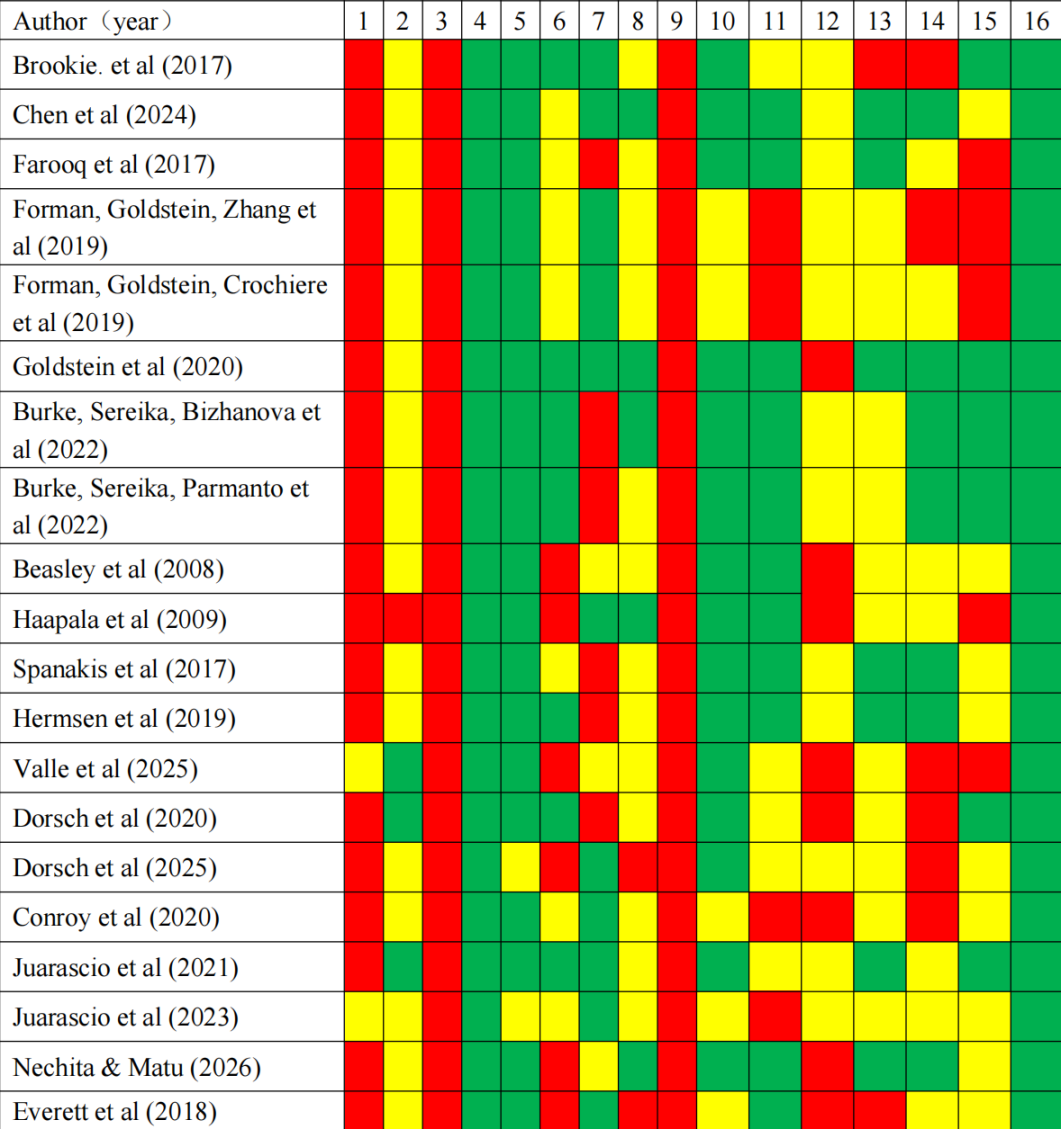
Mobile Health Evidence Reporting and Assessment (mERA) reporting quality assessment of included just-in-time adaptive intervention (JITAIs) studies in dietary health management. Green: fully reported; yellow: partially reported; red: not reported. mERA Project Description: 1. Clearly present the availability of infrastructure supporting technology operation at the study site. 2. Describe and justify the rationality of the technical architecture. 3. Explain how the mHealth intervention integrates with existing health information systems. 4. Clearly describe the implementation method of the mHealth intervention. 5. Detail the content specifics of the intervention, including content sources and any modification explanations. 6. Elaborate on formative research, content testing, or usability testing targeting specific target groups (if applicable). 7. Describe user feedback or satisfaction evaluations of the intervention. 8. Mention barriers or facilitators to study participants’ adoption of the intervention. 9. Present a basic cost assessment of the mHealth intervention from different perspectives. 10. Describe how personnel are informed about the project (including relevant training). 11. Clearly present the limitations of the mHealth solution in large-scale implementation. 12. Describe whether the solution needs to be adjusted for different languages, populations, or contexts. 13. Detail the intervention measures supporting reproducibility. 14. Explain data security procedures/confidentiality agreements. 15. Illustrate the mechanism to ensure that the content or other guidance/information provided by the intervention complies with current national/regulatory guidelines. 16. Was the intervention implemented as planned [28-47].

**Figure 2.**
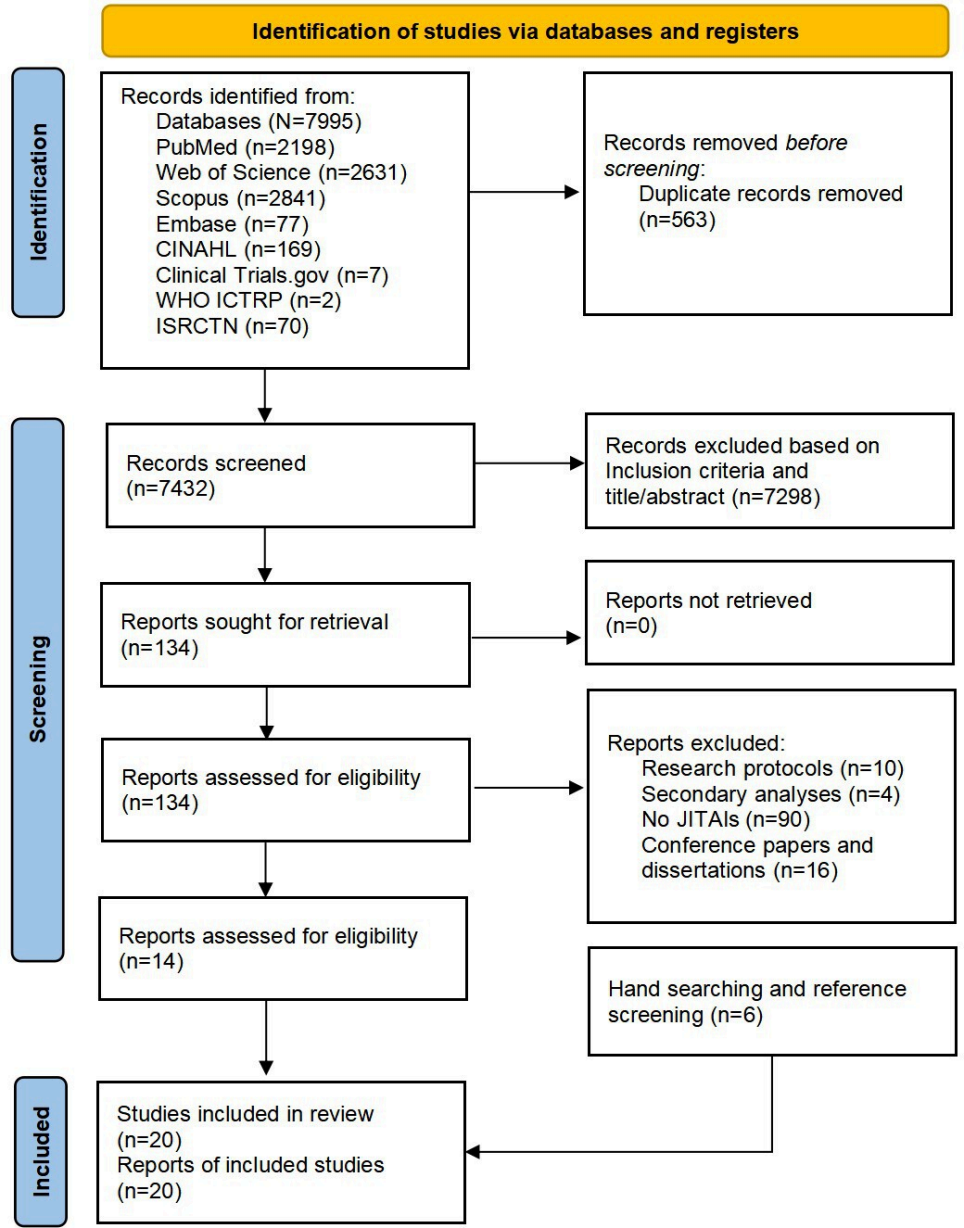
PRISMA (Preferred Reporting Items for Systematic Reviews and Meta-Analyses) 2020 flow diagram of study selection for just-in-time adaptive interventions (JITAIs) in dietary health management. ISRCTN: International Standard Randomized Controlled Trial Number; WHO ICTRP: World Health Organization International Clinical Trials Registry Platform.

A total of 7995 records were identified from the databases and clinical trial registries. The number of records retrieved from each individual source is presented in Multimedia Appendix 2. After deduplication using EndNote X21 followed by manual verification, 563 duplicate records were removed, leaving 7432 records for title and abstract screening. Of these, 134 articles proceeded to full-text eligibility assessment, and 120 were ultimately excluded. Reasons for exclusion included ineligible intervention, inappropriate study design, no reporting of relevant outcomes, study protocol only, or insufficient full text and data for extraction. In addition to electronic searches, backward citation tracing was performed on the reference lists of included studies and relevant systematic reviews, yielding 6 additional eligible studies. The detailed results of mERA reporting quality assessment of included JITAI studies in dietary health management are shown in Figure 1 and the study selection process is shown in Figure 2. The database search strategies are provided in Multimedia Appendix 1.

### Study Characteristics

Among the 20 included studies, 11 [28-37,45] were RCTs, whereas the remaining 9 [38-44,46,47] were nonrandomized studies. Fifteen studies were conducted in the United States [29,30,33-43,45,47], 2 in the Netherlands [32,44], 1 in Finland [31], 1 in New Zealand [28], and 1 in Romania [46]. The sample sizes varied substantially, ranging from 5 participants [42] to 602 participants [34]. Eight studies [29-31,37,40,41,46,47] reported participants’ educational level, 6 [29,30,37,40,41,47] reported economic or occupational status, and 16 [28-30,33-38,40-43,45-47] provided information on participants’ race or ethnicity. Seventeen studies [28-38,40,41,43,45-47] clearly described their recruitment methods. The target populations of the included studies could be categorized into 4 groups: individuals with overweight or obesity (n=10) [29-32,35-38,40,43], individuals with chronic diseases (n=4) [33,34,41,47], individuals with eating disorders (n=3) [42,45,46], and general population groups engaged in dietary management (n=3) [28,39,44]. The basic characteristics of the included studies are presented in Table S2 in Multimedia Appendix 4.

### Quality Assessment of the Studies Included

According to the MMAT and mERA assessments, the included studies were of generally acceptable methodological and reporting quality. In terms of methodological quality, most RCTs demonstrated relatively rigorous standards within their respective design categories. In contrast, although nonrandomized studies and pilot or feasibility studies provided valuable evidence regarding feasibility, acceptability, and preliminary effectiveness, they were more susceptible to limitations related to confounding, between-group comparability, and completeness of outcome reporting. The mERA assessment further indicated that most studies reported the intervention platform, core functions, and implementation procedures with reasonable clarity; however, reporting remained incomplete in areas such as interoperability, data security and privacy, cost, system maintenance, and real-world implementation. No studies were excluded on the basis of quality assessment; instead, methodological quality and reporting completeness were taken into account when interpreting the review findings.

Given the substantial heterogeneity across the included studies in terms of populations, study designs, intervention formats, and outcome measures, the findings were compared primarily through narrative synthesis rather than statistical pooling. Overall, more consistent positive findings were observed for behavioral outcomes, whereas the results for clinical and physiological outcomes were relatively more heterogeneous.

### JITAIs Features

Through these 20 studies, the core characteristics of JITAIs in dietary health management were systematically synthesized (Table S3 in Multimedia Appendix 4).

The 20 included studies involved 16 distinct JITAIs interventions: OnTrack [29,35,43], SMARTER [36,37], CBT+ [42,45], LowSalt4Life [33], myBPmyLife [34], sipIT [41], Sweetch [47], ThinkSlim [44], Nudge app [40], augmented fork with vibrotactile feedback [32], Automatic Ingestion Monitor [39], mobile phone–operated weight-loss program [31], DietMatePro [30], mobile phone–based EMI for fruit and vegetable consumption [28], smartphone-delivered self-compassion EMI [46], and instrumented companion app paired with FatSecret [38]. Table S3 in Multimedia Appendix 4 summarizes the key features of these interventions, including their triggering logic, delivery methods, and the technological platforms on which they relied.

With regard to triggering mechanisms, one study [31] used only user-initiated triggering, 7 studies [28,32,33,38-40,47] used only system-initiated passive triggering, and 12 studies [29,30,34-37,41-46] combined active and passive triggering. Active triggering primarily referred to situations in which users initiated the intervention process themselves, such as by sending an SMS text message and subsequently receiving tailored feedback from the system [31]. Passive triggering generally referred to the automatic delivery of interventions based on prespecified time points, randomized schedules, sensor data, or contextual information. Examples included fixed-time message delivery [28], context-sensitive notifications based on time preferences or activity transitions [38], geolocation-based contextual prompts [33], fully automated personalized messaging based on smartphone data [47], real-time feedback triggered by device-detected eating behavior [32,39], and intervention messages delivered at microrandomized decision points [40]. Combined active and passive triggering typically involved users first completing self-monitoring [30,36,37,41], ecological momentary assessment [29,35,43,44], symptom or emotion reporting, or goal setting [42,45,46], after which the system automatically assessed risk and delivered JITAIs feedback, as well as further personalized notifications or risk alerts based on user-input data [34].

### Effectiveness

Overall, JITAIs-based digital dietary health interventions demonstrated some positive effects across the included studies, although the findings were marked by substantial heterogeneity. More consistent benefits were observed primarily in behavioral and process-related outcomes, including dietary adherence, fruit and vegetable intake, sodium-restriction behaviors, drinking automaticity, self-monitoring performance, eating rate, and notification responsiveness [28,30,32,33,38,41]. In contrast, findings for distal clinical outcomes, such as body weight, blood pressure, HbA_1c_, and clinical symptoms of eating disorders were less consistent. Some studies reported improvements, whereas others did not find significant incremental effects of the intervention compared with the control condition [29,31,34,36,42,45,47]. Across population groups, the effects of JITAIs varied. Among individuals with overweight or obesity, intervention effects were primarily reflected in dietary behavior regulation, self-monitoring, and improvements in some short-term weight-related indicators, whereas evidence for sustained long-term weight loss benefits remained inconsistent [29,30,32,36,43]. In populations with chronic conditions, positive findings were more commonly observed in behavioral changes, such as improvements in water intake, sodium restriction, and physical activity, as well as in certain intermediate outcomes, while effects on primary clinical end points were relatively limited [33,34,41,47]. In populations with eating disorders, some studies suggested that JITAIs may facilitate skill use, alleviate certain symptoms, and buffer maladaptive behaviors following high-risk emotional states; however, their incremental therapeutic effects still require confirmation through more RCTs [42,45,46]. Studies targeting dietary management in the general population indicated that JITAIs may have potential to promote fruit and vegetable intake, reduce meal-level intake, and enhance real-time dietary recording behaviors [28,38,39]. Overall, the main advantage of JITAIs appears to lie in providing timely behavioral support at critical moments, whereas their sustained impact on clinical outcomes remains to be established by further high-quality research.

## Discussion

### Overview

This systematic review suggests that the potential value of JITAIs in dietary health management lies primarily in their ability to support immediate behaviors and process-related outcomes, whereas their effects on distal clinical outcomes remain inconsistent. This indicates that JITAIs may be better understood as a behavioral regulation framework that provides support during moments of elevated risk or vulnerability, rather than as a stand-alone intervention capable of directly producing clinical improvement independent of behavioral processes. Existing studies have covered a range of contexts, including individuals with overweight or obesity, chronic diseases, eating disorders, and dietary management in the general population, with diverse delivery modalities and triggering mechanisms. However, the observed differences in effects across populations, technological approaches, and outcome levels suggest that the effectiveness of JITAIs is likely shaped by the combined influence of target behaviors, triggering precision, user engagement, and implementation context.

### JITAIs in the Context of Dietary Health Management Outcomes

Overall, the effects of JITAIs in dietary health management cannot be captured by a single overarching conclusion, largely because of the substantial heterogeneity among the included studies [20-22]. Different studies targeted different behavioral goals: some focused on weight loss–related behaviors achieved through dietary intervention, whereas others emphasized sodium reduction, water intake, fruit and vegetable consumption, or the management of symptoms in individuals with eating disorders. In addition, marked differences existed across studies in the baseline risk of the target populations, control conditions, follow-up duration, and analytic approaches. Thus, even though all studies were categorized under dietary health management, their actual intervention content and levels of outcomes were not uniform [28,31,33,41,45]. Under these circumstances, simply classifying all studies as either “effective” or “ineffective” would risk obscuring the true characteristics of the field. Nevertheless, one relatively clear pattern can still be observed: JITAIs appear more likely to influence proximal behavioral and process-related outcomes before affecting more distal clinical outcomes [48,49].

This pattern is broadly consistent with the theoretical framework of JITAIs. The core value of JITAIs lies not merely in increasing the frequency of prompts but in delivering support that is matched to an individual’s current state, based on real-time data, at moments of heightened risk, vulnerability, or receptivity to intervention [12,18,50]. Dietary behavior itself is highly context-dependent and is shaped by the combined influence of time, location, emotion, social environment, prior behavior, and immediate cues [51-53]. In such a behavioral context, JITAIs, compared with traditional digital interventions delivered at fixed times with standardized content, are more likely to provide support when behavioral deviation first emerges. As a result, they may be particularly well-suited to influencing proximal behaviors such as fruit and vegetable intake, low-sodium choices, water consumption, self-monitoring, regular eating patterns, and the practice of therapeutic skills [12,33,34,41,54]. By contrast, distal outcomes such as body weight, blood pressure, or glycemic control generally require a longer period of cumulative behavioral change before measurable improvement can occur, making it more difficult to demonstrate stable and consistent incremental effects under these study conditions [20-22]. Thomas Craig et al [55] noted that JITAIs have shown promise in improving health behaviors such as physical activity and dietary behavior but that their long-term effects remain difficult to determine because most included studies had relatively short intervention durations. Koh et al [54] similarly pointed out that although some studies reported improvements in body weight, BMI, waist circumference, and blood pressure, the field as a whole remains at an early stage, with considerable heterogeneity in outcomes, delivery methods, and data collection approaches. In other words, under current research conditions, JITAIs may be better understood as supportive tools that act on behavioral processes, rather than as standalone therapeutic approaches capable of directly and consistently improving body weight, blood pressure, or metabolic indicators [56,57]. This interpretation is also supported by the levels of outcomes reported in the included studies. However, this does not necessarily mean that the interventions are ineffective. Rather, it more likely suggests that proximal behavioral outcomes are more sensitive to contextualized support, whereas distal outcomes require longer-term behavioral accumulation and are also jointly influenced by baseline treatment intensity, sample differences, and multiple behavioral pathways [12]. Therefore, if the value of JITAIs in dietary health management is judged solely on the basis of distal clinical indicators, their practical contribution to behavior enactment, behavior maintenance, and intervention in high-risk situations may be underestimated.

### Patterns Across Target Populations

Across target populations, the application focus of JITAIs shows clear population-specific heterogeneity. Among individuals with overweight or obesity, these interventions have been used primarily to reduce loss-of-control eating, improve dietary adherence, support weight-management behaviors, and maintain self-monitoring [29-32,35-38,40,43]. In populations with chronic conditions, interventions have placed greater emphasis on sodium restriction, water intake, physical activity, and disease-related lifestyle management, reflecting a strong self-management support function [33,34,41,47]. Among individuals with eating disorders, JITAIs have more often served as an extension of therapy by reinforcing skill use and coping strategies during moments of high-risk emotion, intense urges, or symptom vulnerability [42,45,46]. In studies involving the general population, the primary focus has been on promoting fruit and vegetable intake, modifying eating behaviors, and improving the timeliness of dietary recording [28,39,44]. These differences suggest that JITAIs should not be understood as a uniform technology that is equally effective for all dietary health management problems. Rather, they may be better conceptualized as an intervention framework that can be adapted in real time according to the target behavior, risk mechanism, and context of use [11,12,20]. This feature also has important implications for future research. In designing JITAIs, future studies should move beyond simply asking whether a JITAI is used and instead clarify the specific behavioral problem that the JITAIs are intended to address. For example, in populations with overweight or obesity, the key objectives may be to prevent loss-of-control eating and sustain long-term self-monitoring, whereas in populations with chronic diseases, the priority may be to support disease-related lifestyle behaviors. In other words, JITAIs across different populations should not seek formal uniformity, but rather should be designed differentially according to behavioral targets and clinical contexts.

### Technology Usability

Technological implementation approaches were also heterogeneous, encompassing both machine learning–based algorithms [29,35,43,44,47] and rule-based logic driven by prespecified rules, thresholds, or contextual variables [30,32-34,36-39,41,42,45,46]. Current evidence remains insufficient to determine whether any particular algorithmic approach offers a general advantage in dietary health management. However, the included studies suggest that intervention performance is shaped less by algorithmic complexity per se than by whether the intervention can deliver sufficiently useful, contextually appropriate, and acceptable support at the right moment [17-19,50]. Some studies improved notification responsiveness, timeliness of self-monitoring, or app engagement without producing corresponding significant improvements in clinical outcomes, indicating that the value of JITAIs is closely related to triggering precision, user burden, device compatibility, and alignment with the target behavior [29,34,38,45]. Across the included studies, diet-related behaviors were more often assessed through self-monitoring, ecological momentary assessment, or semiautomated sensing, whereas behaviors such as activity level or eating rate were more readily captured continuously by devices [30,32,35,47]. This suggests that JITAIs are more likely to exert their initial effects on behavior enactment, behavior maintenance, and other proximal processes and only subsequently influence distal health outcomes over a longer time scale. Accordingly, JITAIs may be better understood as behavior support systems embedded in real-world contexts, rather than as stand-alone therapeutic approaches capable of producing direct and stable clinical improvements independently of behavioral processes [20,35,42]. From a technological implementation perspective, JITAIs-based dietary health management has already demonstrated a certain degree of feasibility and usability, with diverse formats capable of reaching multiple populations and application scenarios [31,33,41,45,47]. These technological approaches enable JITAIs to deliver personalized interventions in homes, communities, restaurants, supermarkets, and other everyday settings, suggesting potential applications in community health management, outpatient follow-up for chronic disease, weight management programs, and extended care for eating disorders.

### Limitations

This review has several limitations. First, because no consensus definition of JITAIs has yet been established, the terminology used in this field remains inconsistent, which posed challenges for study screening and inclusion and may have led to the omission or misclassification of relevant studies. Second, this review searched only biomedical databases and did not include literature from fields such as human-computer interaction and digital health technology, which may have resulted in incomplete coverage of relevant evidence. In addition, intervention effects may be influenced by digital literacy levels of participants, as some individuals may experience difficulties in operating devices. The implementation of certain JITAIs interventions may also be constrained by device or software incompatibility, while signal limitations in mobile devices may introduce data bias for some participants, resulting in unequal intervention accessibility and affecting the generalizability of the findings. Finally, this review adopted a narrative synthesis rather than a meta-analysis. Although this approach was more appropriate for addressing the substantial heterogeneity across studies in terms of target populations, intervention formats, and outcome measures, it also limited the ability to quantitatively compare effect sizes and formally assess the certainty of the evidence.

### Conclusions

This review systematically synthesized the current applications and intervention effects of JITAIs in dietary health management. Compared with previous reviews, this study makes a novel contribution by specifically focusing on dietary health management as a distinct behavioral domain and by disentangling differences in intervention characteristics, technological delivery modes, and effectiveness across diverse populations, thereby providing a targeted synthesis for this field. The review clarified the application of JITAIs across 4 population groups and further identified existing challenges, including substantial heterogeneity and inconsistent reporting within the literature. These findings not only offer guidance for the standardized design of future JITAI interventions and the conduct of high-quality research but also provide a foundation for the implementation of personalized dietary behavior interventions in clinical and public health settings. In addition, the conclusions of this review may inform the development of precision digital intervention systems in contexts such as community health management, outpatient follow-up for individuals with chronic disease, and early intervention for individuals with eating disorders, thereby facilitating the translation of JITAIs technology from research into scalable and practical public health strategies to improve population dietary health.

## Supplementary material

10.2196/92139 Multimedia Appendix 1 Search strategies for databases and registries.

10.2196/92139 Multimedia Appendix 2 Number of records identified from each information source.

10.2196/92139 Multimedia Appendix 3 Mixed Methods Appraisal Tool assessment results.

10.2196/92139 Multimedia Appendix 4 Research information related to just-in-time adaptive interventions for dietary health management.

10.2196/92139 Checklist 1 PRISMA checklist.

10.2196/92139 Checklist 2 PRISMA-S checklist.

## Abbreviations

EMI: ecological momentary intervention
GRADE: Grading of Recommendations Assessment, Development and Evaluation
JITAIs: just-in-time adaptive interventions
mERA: Mobile Health Evidence Reporting and Assessment
MMAT: Mixed Methods Appraisal Tool
PICOS: population, intervention, comparator, outcomes, and study design
PRISMA: Preferred Reporting Items for Systematic Reviews and Meta-Analyses
PRISMA-S: Preferred Reporting Items for Systematic Reviews and Meta-Analyses—Literature Search Extension
PROSPERO: International Prospective Register of Systematic Reviews
RCT: randomized controlled trial
WHO: World Health Organization
