# Supplementary material for: Application of Just-in-Time Adaptive Interventions in Dietary Health Management: Systematic Review
Source: J Med Internet Res. 2026 Jul 16;28:e92139. doi: 10.2196/92139 (PMC13374797; doi:10.2196/92139)
Supplement: Multimedia Appendix 4 [file jmir-v28-e92139-s004.docx]

Table2. Study and participant characteristics of included JITAIs studies in dietary health management.

| Researchers, year, and country | Study Design | Study Population, Inclusion Criteria and Research Setting | Sample size and recruitment method | Characteristics of the study subjects (age, gender, ethnicity, educational level, occupation and socioeconomic status) |
| --- | --- | --- | --- | --- |
| Brookie et al^[[34](file:///C:/Users/HP/Desktop/eeeee888.docx#_nebF271D7AE_8647_4F42_A326_0111F1BCA680)]^，2017, New Zealand | RCT | Young adults with low fruit and vegetable intake.  Inclusion criteria: aged 18–25 years; ownership of an internet-enabled smartphone; daily fruit and vegetable intake of ≤3 servings; not currently taking antidepressant medication; and no history of fruit or vegetable allergy.  Exclusion criteria: none explicitly specified. Setting: university-based setting in Dunedin, New Zealand. | n=171, recruited through a student employment agency (21%) and undergraduate psychology courses (79%). | Age: 19.4 ± 1.45 years. Sex: 67.3% female. Ethnic composition: 64.3% European, 18.0% Asian, 8.0% Māori/Pacific Islander, and 11.0% other or mixed ethnicity. Educational and socioeconomic characteristics: not reported. |
| Chen et al^[[45](file:///C:/Users/HP/Desktop/eeeee888.docx#_neb531E37EA_D145_4B7F_AD06_746700871C23)]^. 2024 USA | Non-randomized study | Adults from the general population aged 18–70 years. Inclusion criteria: 18–70 years of age; ownership of a Samsung Android smartphone; access to a weighing scale (provided free of charge by the research team if unavailable). Setting: free-living, everyday environment. | n=30, recruited through word-of-mouth referrals, university mailing lists, posted flyers, and in-person recruitment at local community venues, including libraries and museums. | Age: 27.93 ± 8.47 years. Sex: 66.7% male (n = 20) and 33.3% female (n = 10). Ethnicity: 63.3% Asian, 16.7% White, 6.7% Black/African American, 3.3% Hispanic/Latino, 6.7% Middle Eastern, and 3.3% preferred not to answer. Educational attainment: not reported. Socioeconomic and occupational characteristics: not reported. |
| Farooq et al^[[44](file:///C:/Users/HP/Desktop/eeeee888.docx#_neb25693FFD_BE29_436B_8D37_EAEAFB7FC9B6)]^. 2017, USA | Non-randomized study | Adults. Inclusion criteria: not described in detail. Setting: The University of Alabama. | n=18. The recruitment method was not reported. | Age: 27.7 ± 2.8 years. Sex: 15 male participants (83.3%) and 3 female participants (16.7%). Ethnicity: not reported. Educational attainment: not specified. Socioeconomic and occupational characteristics: not specified. |
| Forman et al^[[33](file:///C:/Users/HP/Desktop/eeeee888.docx#_neb9333FAAE_F44C_4A66_AFF8_DA8B035A895E)]^，2019. USA | RCT | Adults with overweight or obesity. Inclusion criteria: ownership of an iPhone with an active data plan; willingness to purchase or borrow a wireless scale; BMI of 25–50 kg/m²; age 18–70 years; and residence in the United States. Exclusion criteria: ≥5% recent weight loss; current participation in another structured weight-management program; history of bariatric surgery; planned pregnancy during the study period; presence of eating disorder symptoms; changes in weight-affecting medications within the previous 3 months; or any serious medical condition affecting body weight, appetite, or eating behavior. Setting: community-based setting. | n=181, recruited through press releases, Facebook advertisements, and radio advertisements. | Mean age: 46.29 ± 13.58 years. Sex: 85.1% female. Race/ethnicity: 73.5% White, 12.2% African American/Black, 9.4% Hispanic, 3.3% Asian/Pacific Islander, 2.2% Asian American, 1.7% African, 1.7% European, 0.6% Native American/American Indian, and 3.3% other. Educational and socioeconomic characteristics: not reported. |
| Forman et al^[[46](file:///C:/Users/HP/Desktop/eeeee888.docx#_nebEBFCC840_F13D_48FE_83C7_CD81AC8DA621)]^，2019. USA | Non-randomized study | Adults with overweight or obesity aged 18–65 years (BMI 25–50 kg/m²) who owned an iPhone. Exclusion criteria: participation in another structured weight-loss program; current pregnancy or plans to become pregnant; self-reported eating disorder symptoms; any medical condition contraindicating weight loss; dose changes within the past 3 months in medications affecting body weight or appetite; and a history of bariatric surgery. Setting: community-based setting. | n=43, recruited through print and online advertisements and underwent a brief telephone screening. | Mean age: 50.98 ± 12.72 years. Sex: 86.0% female (37 women, 6 men). Race/ethnicity: 74.4% White, 18.3% Black, 2.3% Hispanic/Latino, and 4.6% other. Educational and socioeconomic characteristics: not reported. |
| Goldstein et al^[[38](file:///C:/Users/HP/Desktop/eeeee888.docx#_neb4B3FA180_902A_4EF2_96BD_E9AF4DBC933C)]^, 2020, USA | RCT | Adults residing in the United States, aged 18–70 years, with overweight or obesity (BMI 25–50 kg/m²), who were seeking weight loss treatment and were able to competently use an iOS mobile device. Participants had no eating disorders, no medical contraindications to weight loss, were not enrolled in another structured weight-loss program, and were able to complete the entire remote study protocol. Inclusion criteria: 18–70 years of age and residing in the United States on a long-term basis; BMI of 25–50 kg/m²; ownership of an iOS mobile device with an active data plan and sufficient proficiency in its use; willingness to purchase or borrow a wireless Bluetooth scale; stable use status of medications affecting body weight or appetite for at least 3 months; willingness to provide informed consent and to participate in online screening, intervention, and follow-up procedures. Study setting: United States; fully conducted remotely. | n=121, recruited through multiple public recruitment channels, including mailing lists, press releases, radio advertisements, and social media advertisements. | Sex: 102 female participants (84.3%) and 19 male participants (15.7%). Race/ethnicity: 89 White participants (73.6%), 14 Black/African American participants (11.5%), 7 Asian/Pacific Islander participants (5.8%), and 11 participants from other racial/ethnic groups (9.1%). Educational attainment: the specific distribution of educational levels was not directly reported. Employment and socioeconomic characteristics: 85 participants (70.2%) were employed full-time, 16 (13.2%) part-time, 12 (9.9%) reported no income or flexible/irregular employment, and 2 (1.7%) relied on disability benefits or social assistance. |
| Burke et al^[[30](file:///C:/Users/HP/Desktop/eeeee888.docx#_nebF57F5016_F528_4379_921D_2D2EBA34027F)]^. 2022, USA | RCT | Adults with overweight or obesity. Inclusion criteria: age ≥18 years; BMI 27.0–43.0 kg/m²; regular use of a smartphone with an active data plan; ability to engage in moderate-intensity physical activity; and ability to complete a 5-day electronic food diary with a recorded daily energy intake of ≥700 kcal/day. Setting: community-based settings in and around Pittsburgh, Pennsylvania, USA, combined with a remotely delivered mobile-device–supported intervention. | n=502, recruited from communities in the greater Pittsburgh area of Pennsylvania, United States. | Age: 45.0 ± 14.4 years. Sex: 79.5% female (n = 399) and 20.5% male (n = 103). Race/ethnicity: 82.5% White, with the remainder identifying as non-White. Educational attainment: not reported. Socioeconomic and occupational characteristics: not reported. |
| Burke et al^[[31](file:///C:/Users/HP/Desktop/eeeee888.docx#_nebBBF9B20D_99F7_4852_93C3_BDCF8651FA5A)]^. 2021, USA | RCT | Adults with overweight or obesity. Inclusion criteria: age ≥18 years; BMI 27.0–43.0 kg/m²; regular use of a smartphone with an active data plan; ability to engage in moderate-intensity physical activity; and ability to complete a 5-day electronic food diary with a recorded daily energy intake of ≥700 kcal/day. Setting: community-based settings in and around Pittsburgh, Pennsylvania, USA, combined with a remotely delivered mobile-device–supported intervention. | n=502, recruited from communities in the greater Pittsburgh area of Pennsylvania, United States. | Age: 45.0 ± 14.4 years. Sex: 79.5% of participants were female (n = 399), and 20.5% were male (n = 103). Race/ethnicity: 423 participants (84.3%) were White, and 79 (15.7%) were non-White. Educational attainment: mean years of education was 16.4 ± 2.8 years. Socioeconomic and employment characteristics: 82.1% of participants were employed either full-time or part-time (n = 412), whereas 17.9% were not employed (n = 90), including those who were unemployed, retired, or disabled. Regarding household income, 62.4% of participants reported an annual household income of ≥US$60,000 (n = 313), 28.5% reported <US$60,000 (n = 143), and the remainder had missing income data. |
| Beasley et al^[[29](file:///C:/Users/HP/Desktop/eeeee888.docx#_neb5907E13E_89E3_4716_8E51_D575021407C7)]^. 2009, USA | RCT | Adults with overweight or obesity (BMI 25–40 kg/m²) and no dietary restrictions. Inclusion criteria: BMI of 25–40 kg/m²; computer literacy (defined as using a computer at least three times per week); ability to read the Palm Zire 21 screen; and physician approval to participate in a very-low-fat vegetarian dietary intervention. Exclusion criteria: currently following a special diet for medical reasons. Setting: Personal Improvement Computer Systems, Inc., Reston, Virginia, USA. | n=147, recruited through an advertisement placed in the health section of The Washington Post. | Sex: women comprised the majority of participants (83% in the intervention group and 77% in the control group). Race/ethnicity: Intervention group: 85% White, 10% Black, and 5% Asian. Control group: 83.3% White and 16.7% Black. Educational attainment: participants had a mean of approximately 17 years of education. Socioeconomic characteristics: not directly reported; however, most participants were employed full-time (39% in the intervention group and 52% in the control group). |
| Haapala et al^[[28](file:///C:/Users/HP/Desktop/eeeee888.docx#_neb2C6756B1_FACB_4A87_A65C_B22BCC20C895)]^. 2009, Finland | RCT | Healthy adults with overweight (BMI 26–36 kg/m²), aged 25–44 years. Inclusion criteria: BMI of 25–36 kg/m²; age 25–44 years; ownership of a mobile phone and access to an internet connection. Setting: University of Kuopio, Kuopio, Finland. | n=125, recruited through newspaper advertisements and screened by telephone interview. | Sex: women comprised the majority of participants (79% in the intervention group and 76% in the control group). Race/ethnicity: not reported. Educational attainment: Intervention group: 23% had a postgraduate degree, 44% had a bachelor’s degree, and 10% had a vocational secondary education. Control group: 18% had a postgraduate degree, 50% had a bachelor’s degree, and 11% had a vocational secondary education. Socioeconomic characteristics: not reported. |
| Spanakis et al^[[43](file:///C:/Users/HP/Desktop/eeeee888.docx#_nebBECB419B_0740_4AE5_AEB4_34B70CAF4482)]^. 2017, Netherlands | Non-randomized study | Adults with overweight or obesity. Inclusion criteria: ability to use a smartphone and willingness to complete EMA data collection over a 2–8 week period. In Study I, participants included both adults with overweight and healthy-weight adults; in Study II, only adults with overweight were included. All participants were required to have basic smartphone literacy. Setting: the naturalistic daily-life environment in and around Maastricht University, the Netherlands. | n=200. The recruitment method was not explicitly reported. | Details were not reported for either study. |
| Hermsen et al^[[35](file:///C:/Users/HP/Desktop/eeeee888.docx#_neb48E91B0C_6BD9_40EE_9F2F_D8F72D448E41)]^. 2019， Netherlands | RCT | Adults with overweight or obesity. Inclusion criteria: Age ≥18 years; self-identified fast eaters; body mass index (BMI) ≥25 kg/m². Setting: participants’ natural eating environment. | n=141, recruited by dietitians from 30 dietary practice centers, who enrolled participants from among their existing clients. | Age: 49.2 ± 12.3 years. Sex: NFC control group (questionnaire sample, n = 36): 58% female and 42% male. VFC vibration feedback group (questionnaire sample, n = 44): 59% female and 41% male. VFC + vibration plus visual feedback group (questionnaire sample, n = 34): 65% female and 35% male. Race/ethnicity: not reported in detail, although participants were explicitly recruited primarily from local communities in the Netherlands. Educational attainment: not specified. Socioeconomic and occupational characteristics: not specified. |
| Valle et al^[[42](file:///C:/Users/HP/Desktop/eeeee888.docx#_nebFDF6038E_AED5_434B_A5CB_E62D45311BBC)]^. 2025, USA | Non-randomized study | Young adults aged 18–35 years with a body mass index (BMI) of 25–40 kg/m². Inclusion criteria: self-reported moderate-to-vigorous physical activity of <150 minutes per week; ownership of an iPhone (iOS 11.0 or later); and access to a wireless network compatible with the smart scale. Exclusion criteria: pregnancy or plans to become pregnant; night-shift work; any health condition that could interfere with dietary or physical activity modification; and participation in another weight-control program. Setting: community-based setting. | n=52, recruited through university mailing lists and paid Facebook advertisements, followed by online screening, telephone follow-up, and a group enrollment session. | Age: 29.6 ± 3.8 years. Sex: 79.2% female (42/53) and 20.8% male (11/53). Race/ethnicity: 62.3% White (33/53), 16.9% African American/Black (9/53), and 20.8% other or multiracial (11/53). Educational attainment: 84.9% had a college degree or above, including 25 participants with a bachelor’s degree and 20 with a master’s or doctoral degree. Socioeconomic characteristics: 32.1% reported an annual income of US$50,000–74,999, and 17.0% reported an annual income >US$100,000. |
| Dorsch et al^[[32](file:///C:/Users/HP/Desktop/eeeee888.docx#_neb9CEC5D77_1796_41F8_B5A1_3C6D4A749FE1)]^. 2020, USA | RCT | Adults with hypertension (≥18 years of age). Inclusion criteria: receipt of antihypertensive treatment for at least 3 months and use of an iPhone. Exclusion criteria: chronic kidney disease (CKD), heart failure, systolic blood pressure >180 mmHg, diastolic blood pressure >110 mmHg, insulin-dependent diabetes, and current use of loop diuretics, corticosteroids, or nonsteroidal anti-inflammatory drugs (NSAIDs); baseline sodium intake ≥2000 mg/day (with subsequent adjustment). Setting: Michigan Medicine. | n=50, recruited through a university-based recruitment platform and by mailing invitation letters to more than 7,000 potentially eligible patients. | Age: 56.6 ± 10 years in the intervention group and 58.2 ± 11 years in the control group. Sex: 58% female in the intervention group and 61% female in the control group. Race/ethnicity: Intervention group: 79% White, 8% African American, 8% Asian, and 4% other. Control group: 81% White, 12% African American, 4% Asian, and 4% other. Educational attainment: not explicitly reported. Socioeconomic characteristics: not explicitly reported. |
| Dorsch et al^[[36](file:///C:/Users/HP/Desktop/eeeee888.docx#_neb2D368451_5A6D_43C0_B61F_A5B2558A5ACD)]^，2025 , USA | RCT | Patients with hypertension. Inclusion criteria: a confirmed diagnosis of hypertension; no changes in antihypertensive medication during the previous 4 weeks; daily sodium intake ≥1500 mg, as assessed using the Block Sodium Screener; and ownership of a compatible smartphone (iOS 12.0+ or Android 8.0+). Exclusion criteria: any contraindication to physical activity or to a low-sodium diet. Setting: clinical settings at the University of Michigan Health System and the Hamilton Community Health Network, USA. | n=602, recruited through email, text messaging, and telephone contact. | Age: 59.6 ± 13.6 years. Sex: 48.0% female. Race/ethnicity: 74.4% White, 13.0% Black, 9.5% Asian, and 2.2% other. Educational attainment and socioeconomic characteristics were not reported. |
| Conroy et al^[[41](file:///C:/Users/HP/Desktop/eeeee888.docx#_nebA8CEC2EB_8D81_4C71_A6BB_D4A5471A9046)]^，2020, USA | Non-randomized study | Patients with a history of kidney stones. Inclusion criteria: age ≥18 years; fluency in spoken and written English; ability to provide informed consent; ownership of an iPhone (iOS 7 or later); and no fluid intake restrictions. Setting: community-based setting. | n = 31, recruited through advertisements placed in community settings and urology clinics. | Age: 40.0 ± 14.3 years. Sex: 58% female. Race/ethnicity: 97% were non-Hispanic, and 87% were White. Employment status: 61% were employed full-time. Educational attainment: the median educational level was a bachelor’s degree. |
| Juarascio et al^[[40](file:///C:/Users/HP/Desktop/eeeee888.docx#_neb8A8E9441_404E_4E58_B2E7_3F9DC2DF417D)]^， 2021, USA | Non-randomized study | Patients with bulimia nervosa (BN). Inclusion criteria: 18–70 years of age and meeting the primary diagnostic criteria for BN. Exclusion criteria: severe psychiatric comorbidity (e.g., psychotic disorders, acute suicidal ideation, or severe substance use disorder); inability to speak or write English; intellectual impairment that would interfere with app use; and prior completion of a full course of cognitive behavioral therapy (CBT) for BN. Setting: a clinical setting at the Center for Weight, Eating, and Lifestyle Science, Drexel University, USA. | n=5. The recruitment method was not explicitly reported. | Age: 35.6 ± 6.8 years. Sex: 4 female participants and 1 male participant. Race/ethnicity: 4 participants self-identified as White, 1 as multiracial, and 2 as Latino/Hispanic. Educational and socioeconomic characteristics: not reported. |
| Juarascio et al^[[37](file:///C:/Users/HP/Desktop/eeeee888.docx#_neb5DFEB74E_FEDD_45A4_9E42_D49DDCA36C89)]^， 2023, USA | RCT | Patients with BN-spectrum eating disorders (BN-spectrum EDs). Inclusion criteria: meeting the behavioral criteria under DSM-5 for bulimia nervosa (BN) or other specified feeding or eating disorder, bulimia nervosa type (OSFED-BN type); reporting at least 12 binge-eating episodes and 12 compensatory behaviors per month in the recent period; 18–70 years of age; body mass index (BMI) >17.5 kg/m²; ability and willingness to use a smartphone application to complete 16 weeks of self-monitoring of eating behavior and symptoms; and fluency in spoken and written English. Setting: Philadelphia, Pennsylvania, USA, with the study conducted by Drexel University. | n=56, recruited in the Philadelphia area through flyers, radio advertisements, and social media advertisements. | Age: 38.9 ± 14.1 years. Sex/gender: 83.9% female (n = 47), 14.3% male (n = 8), and 1.8% nonbinary (n = 1). Race/ethnicity: 64.3% White, 8.9% Black/African American, 7.1% Asian, 1.8% American Indian/Alaska Native, 19.6% multiracial/other, and 3.8% not reported/preferred not to disclose. Educational attainment and occupational characteristics were not reported. |
| Nechita & Matu^[[47](file:///C:/Users/HP/Desktop/eeeee888.docx#_neb30D91FC2_0F8D_4D53_8ECE_327704C41C0B)]^, 2026, Romania | Non-randomized study | Community-dwelling women with elevated levels of eating disorder symptoms. Inclusion criteria: female sex, age ≥18 years, an Eating Disorder Examination Questionnaire (EDE-Q) score >2.4, and ownership of a smartphone. Setting: participants’ naturalistic daily-life environment. | n=93, recruited through study advertisements disseminated on social media platforms (e.g., Facebook and Instagram) as well as through university-affiliated online communities, targeting eligible women from the community. | Age: 30.3 ± 9.0 years in the intervention group (n = 46) and 26.94 ± 7.96 years in the control group (n = 47). Sex: 100% female. Race/ethnicity: all participants were Caucasian/Romanian. Educational attainment: 68.8% held a bachelor’s or master’s degree. Socioeconomic and occupational characteristics: not reported. |
| Everett et al^[[39](file:///C:/Users/HP/Desktop/eeeee888.docx#_nebBC3B9BD4_7D43_4DBC_AA2C_69A3421CAE85)]^. 2018, USA | Non-randomized study | Adults with prediabetes. Inclusion criteria: confirmed prediabetes, defined by meeting at least one of the following criteria: HbA1c 5.7%–6.4%, fasting plasma glucose 100–125 mg/dL, or 2-hour plasma glucose 140–199 mg/dL during a 75-g oral glucose tolerance test (OGTT); BMI 24–40 kg/m² for non-Asian participants and 22–40 kg/m² for Asian participants; ability to communicate in English; ownership of an Android smartphone or an iPhone 5S or later. Setting: the Johns Hopkins Clinical Research Network, centered at the Johns Hopkins University School of Medicine, including The Johns Hopkins Hospital and a community internal medicine clinic in Reading, Pennsylvania. | n=55. Recruitment was conducted through clinical sites within the Johns Hopkins Clinical Research Network, including The Johns Hopkins Hospital affiliated with the Johns Hopkins University School of Medicine and a community internal medicine clinic within the Reading Health System in Pennsylvania. The primary recruitment targets were adults with prediabetes attending primary care and endocrinology outpatient clinics. Additional participants were recruited through multiple supplementary channels, including clinician referral, medical record review, and on-site and social media advertisements. | Age: mean age 57.2 ± 9.1 years. Sex: 24 women (63%) and 14 men (37%). Race/ethnicity: 31 White participants (82%) and 7 Black participants (18%). Educational attainment: 5% had less than a high school education, 18% were high school graduates, 26% had some college education, 3% held an associate degree, 24% held a bachelor’s degree, and 24% had an advanced degree. Employment/socioeconomic characteristics: 55% were employed full-time, 26% part-time, 3% were unemployed, and 15% were retired. |

Table3. Intervention features and main findings of included JITAIs studies in dietary health management.

| Researchers, year, and country | Real-time support: Provision of timing and triggering methods | Types of Data, Software and Hardware Used for Real-Time Support | Duration of intervention | Outcome indicators and measurement methods | Intra-group or inter-group differences |
| --- | --- | --- | --- | --- | --- |
| Brookie et al^[[34](file:///C:/Users/HP/Desktop/eeeee888.docx#_nebF271D7AE_8647_4F42_A326_0111F1BCA680)]^，2017, New Zealand | Based on a dual-trigger framework integrating time-based delivery and participant behavioral characteristics, text messages were delivered twice daily at two fixed time windows (10:00–10:30 during class breaks and 17:00–17:30 before dinner). Message delivery was contingent on the participant having self-reported a fruit and vegetable intake of ≤3 servings on the previous day. If a participant met the target of ≥4 servings of fruit and vegetables for two consecutive days, a reinforcement feedback text message was triggered on the following day. | Data collected: daily fruit and vegetable intake (via smartphone-based questionnaire) and plasma vitamin C and total carotenoid concentrations (as biomarkers). Software/platforms: smartphone questionnaire platform and SMS delivery service. Hardware/devices: smartphone, calibrated weighing scale, and stadiometer. | 2 weeks | Primary outcomes: total daily fruit and vegetable intake (servings/day), as well as daily fruit intake and daily vegetable intake assessed separately. Secondary outcomes: plasma vitamin C concentration; plasma total carotenoid concentration; daily intake of potato chips/French fries and sweet foods (servings/day); and the proportion of participants achieving the recommended intake of ≥5 servings of fruit and vegetables per day. Assessment methods: Fruit, vegetable, and unhealthy food intake was assessed using a 14-day smartphone-based daily online survey. Biomarkers were measured using high-performance liquid chromatography with electrochemical detection (HPLC-ECD) and spectrophotometry. Target attainment was calculated as the proportion of participants meeting the threshold of ≥5 servings/day based on survey data, and between-group differences were compared using the chi-square test. | Intergroup differences: The EMI group (mean 3.7 servings/day) = the FVI group (mean 3.7 servings/day) > the control group (mean 2.8 servings/day) (F (2,167)=14.708, p<0.001). Intragroup differences: Plasma vitamin C levels increased significantly in the EMI group (t (53)=-2.168, p=0.035); plasma carotenoid levels showed an increasing trend in the FVI group (t (54)=-1.994, p=0.051). |
| Chen et al^[[45](file:///C:/Users/HP/Desktop/eeeee888.docx#_neb531E37EA_D145_4B7F_AD06_746700871C23)]^. 2024 USA | The GatorTrack smartphone companion app (integrated with FatSecret) monitored core weight-management data, including body weight, dietary intake, and physical activity, up to five times per day within user-tailored time windows based on individualized daily schedules. When a participant failed to complete the required health data entry within the scheduled time window or at a designated behavioral transition point, and the participant was still within the active notification period (within 60 minutes after push notification delivery), the system immediately triggered a time-based and context-sensitive push notification, including a direct link to the corresponding self-monitoring interface. In parallel, the app backend tracked self-monitoring engagement metrics, including notification click-through rate, data completion rate, and response latency, which were used to support stage-specific follow-up interviews (i.e., midpoint check-in interviews and final user-experience interviews) and to collect participant feedback on the notification experience and potential functional improvements. | Data sources: only two contextual variables were collected, namely user preset schedules and physical activity transitions detected via the Google Activity Recognition Transition API. Responsiveness metrics: notification click-through rate and click response time. Completion metrics: notification completion rate and overall logging rate. No data were collected on geolocation, screen activity, calendar events, weather, or other contextual factors. Software: GatorTrack and FatSecret. Hardware: Samsung Android smartphone and weighing scale. | 4 weeks | Primary outcomes: overall self-monitoring adherence rate, notification click response time, notification click-through rate, and notification completion rate. Secondary outcomes: completion rate of context-triggered notifications, completion rate of timeout-triggered notifications, timeliness of monitoring across different data types, user interaction patterns with notifications, perceived notification effectiveness, and user experience with product functionality. Measurement methods: overall self-monitoring adherence rate, notification click response time, notification click-through rate, notification completion rate, completion rate of context-triggered notifications, completion rate of timeout-triggered notifications, and timeliness of monitoring across different data types were all automatically recorded by the app. User interaction with notifications, perceived notification effectiveness, and experience with product features were assessed through semi-structured interviews. | Intergroup differences:  Notification click response time: The contextualized group (12.33 minutes) was significantly shorter than the temporal group (18.42 minutes), F = 8.2484, p < 0.01;  Notification click rate: The contextualized group (19.05%) was significantly higher than the temporal group (13.96%), F = 7.3207, p < 0.05;  Notification completion rate: The contextualized group (21.77%) was significantly higher than the temporal group (17.32%), F = 7.3831, p < 0.05;  Overall self-monitoring log rate: The contextualized group (58.87%) was slightly higher than the temporal group (55.54%), with no statistical significance (F = 1.1302, p > 0.05). |
| Farooq et al^[[44](file:///C:/Users/HP/Desktop/eeeee888.docx#_neb25693FFD_BE29_436B_8D37_EAEAFB7FC9B6)]^. 2017, USA | An integrated piezoelectric strain sensor was used to continuously monitor temporalis muscle activity in real time to quantify the number of chewing events. When the participant was in an eating episode and the cumulative chew count reached predefined milestones of 25%, 50%, 75%, and 100% of the target value, the system immediately delivered audio feedback via a computer-based interface. In parallel, the sensor system continuously tracked whether the cumulative chew count had reached 100% of the preset target threshold, thereby supporting the triggering of an audio prompt instructing the participant to stop eating. All feedback was delivered synchronously during real-time monitoring of chewing behavior. | Data collected: number of chews, food intake, meal duration, and subjective ratings of hunger and satiety. Software: custom-developed data processing and feedback-generation software based on MATLAB. Hardware: the Automatic Ingestion Monitor (AIM) wearable sensor system and a laboratory laptop computer. | during a single eating episode | Primary outcome: total mass of food consumed. Secondary outcomes: energy intake, meal duration, and changes in multiple subjective ratings assessed by questionnaire. Measurement methods: Food intake (mass consumed): measured using a digital kitchen scale. Energy intake: calculated from the mass of food consumed in combination with the energy density of the meal. Number of chews: measured using the AIM wearable sensor system. Total meal duration and actual chewing duration: recorded using the software accompanying the AIM wearable sensor system. Liquid intake: measured using a digital kitchen scale. Subjective ratings of hunger, satiety, appetite, thirst, and food palatability: assessed using a standard 9-point rating scale. | Quality of food intake: The median intake quality at the 75% target visit (0.431 kg) was significantly lower than that at the baseline visit (0.493 kg), P < 0.05;The median intake quality at the 75% target visit was significantly lower than that at the 100% target visit (0.491 kg), P < 0.05;There was no significant difference in intake quality between the 100% target visit and the baseline visit, P > 0.05. Total meal duration: A significant difference was observed among the three visits (P < 0.05). Post-hoc pairwise comparisons revealed that:The median duration at the 75% target visit was significantly shorter than that at the baseline visit (median change: -21.4%), P < 0.05;The median duration at the 75% target visit was significantly shorter than that at the 100% target visit, P < 0.05;There was no significant difference in duration between the 100% target visit and the baseline visit, P> 0.05. Changes in subjective perception scores: For the pre-meal to post-meal changes in scores of hunger, satiety, desire to eat, expected food intake, thirst, and food palatability, no significant differences were found among the three visits (P > 0.05). |
| Forman et al^[[33](file:///C:/Users/HP/Desktop/eeeee888.docx#_neb9333FAAE_F44C_4A66_AFF8_DA8B035A895E)]^，2019. USA | Using a machine-learning algorithm to analyze 18 real-time variables, the system identified high-risk moments for loss-of-control eating and delivered 1–3 intervention messages tailored to the corresponding risk factors. The first 2 weeks served as a data acquisition period. Beginning in week 3, participants completed six semi-random ecological momentary assessment (EMA) prompts per day; if a participant reported a strong urge to eat (≥7/10) during an EMA survey, an intervention was triggered immediately. | Data collected: episodes of dietary lapse/loss-of-control eating, 18 lapse-triggering factors (e.g., affect, boredom, hunger, and craving), and time-of-day information.  Software: the OnTrack app and the WW app. Hardware: a Yumani Bluetooth-enabled smart scale and an iPhone. | 10 weeks | Primary outcomes: weight change and frequency of dietary lapses. Secondary outcomes: intervention satisfaction, OnTrack engagement/use, and algorithm predictive performance. Measurement methods: Weight change: assessed using a Bluetooth-enabled smart scale. Dietary lapses: in the WW+OT group, these were assessed through six quasi-random EMA prompts per day delivered via OnTrack, supplemented by participant-initiated self-reports. Satisfaction: measured using the Treatment Acceptability and Motivation Scale (TAMS). OnTrack use: automatically recorded by the app based on the number of prompts delivered, prompts completed, app openings, and message views, from which corresponding usage metrics were calculated. Algorithm performance: evaluated by comparing the algorithm’s predictions during weeks 3–8 with self-reported lapse events, with sensitivity and specificity calculated. Generalized linear models (GLMs) were used to control for adherence, and the chi-square test was used to examine the influence of the dietary plan. | Intergroup differences (BTS diet plan): Weight loss in the WW+OT group (mean: 4.7%, standard error: 0.55) was significantly greater than that in the WW-only group (mean: 2.6%, standard error: 0.80) (F [1,173] = 9.68, p = 0.002). Intergroup differences (FS diet plan): Weight loss in the WW-only group (mean: 4.5%, standard error: 0.52) was greater than that in the WW+OT group (mean: 2.9%, standard error: 0.38). Intragroup differences: The frequency of dietary lapses decreased significantly over the 10-week intervention (F [5.49,621.14] = 4.48, p < 0.001). |
| Forman et al^[[46](file:///C:/Users/HP/Desktop/eeeee888.docx#_nebEBFCC840_F13D_48FE_83C7_CD81AC8DA621)]^，2019. USA | The algorithm analyzed 20 variables in real time and triggered a risk alert when a participant reported an urge to eat of ≥6/10 in the presence of highly tempting foods (e.g., potato chips or cake). In addition, if a participant had not logged any eating episode for 3 consecutive hours, the system delivered a regular eating reminder. | Data variables: affect, boredom, hunger, craving, fatigue, food availability, and other temptation-related cues, assessed using ecological momentary assessment (EMA).  Software: an iPhone-based application, formerly known as DietAlert. | 8 weeks | Primary outcomes: frequency of dietary lapse episodes and weight change. Secondary outcomes: app engagement/use, app acceptability and satisfaction, and algorithm predictive performance. Measurement methods: Dietary lapses were recorded in real time using the OnTrack app. Weight change was assessed at baseline and post-intervention using a calibrated scale to measure body weight and a stadiometer to measure height; percentage weight loss and BMI were then calculated. App engagement/use was automatically logged by the app, including the completion rate of six daily surveys, the number of risk alerts received and opened, and the proportion of microinterventions viewed; repeated-measures analysis of variance (ANOVA) was used to examine trends in app use over time. Acceptability and satisfaction were assessed at the end of the intervention using a Technology Acceptance Model (TAM)-based questionnaire to evaluate perceived ease of use, usefulness, and related dimensions. Algorithm performance was evaluated by comparing the algorithm’s predictions with participant self-reported lapse events and calculating the negative predictive value (NPV); participants’ subjective perceptions of the accuracy of the predictions and interventions were also collected through an end-of-study survey. | Mean weight loss was 3.13% (SD = 2.98%; range, −9.00% to 7.00%). The change in BMI from pre- to post-intervention was statistically significant (p = 0.001). Overall, 43.6% of participants achieved ≥3% weight loss, and 35.9% achieved ≥5% weight loss. Unplanned loss-of-control eating decreased significantly (p = 0.02), whereas planned loss-of-control eating did not change significantly (p = 0.29). Weight loss was not significantly associated with loss-of-control eating, either for unplanned episodes (p = 0.52) or planned episodes (p = 0.79). |
| Goldstein et al^[[38](file:///C:/Users/HP/Desktop/eeeee888.docx#_neb4B3FA180_902A_4EF2_96BD_E9AF4DBC933C)]^, 2020, USA | The OnTrack mobile application, used in conjunction with the WW (Weight Watchers) weight-management program and a Bluetooth-enabled smart scale, employed ecological momentary assessment (EMA) to monitor in real time 17 categories of dietary lapse triggers (e.g., affect, hunger, and fatigue) as well as dietary lapse episodes. If, after completion of an EMA survey, the algorithm estimated the risk of a dietary lapse to be ≥40% (low-, moderate-, or high-risk), and the participant was within their usual waking hours, the system immediately delivered a mobile risk-alert notification that included the top three triggering factors and their corresponding coping strategies. Meanwhile, the app backend continuously tracked EMA adherence, risk-alert opening rates, and dietary lapse frequency to support ongoing optimization of the machine-learning algorithm (C4.5 decision tree) and improve its predictive accuracy. The app also allowed participants to self-initiate EMA entries and to access a personalized intervention library containing 157 intervention options. | Data collected: EMA-based data on dietary lapse episodes and 17 categories of triggering factors; body weight data from the Bluetooth-enabled scale; usage data on risk alerts and the intervention library; and demographic data as well as perceived effectiveness ratings.  Software: OnTrack (iOS version), the WW digital weight-loss program, and Weka (C4.5 algorithm).  Hardware: iOS smartphone and Yunmai Bluetooth-enabled smart scale. | 10 weeks | Primary outcome: algorithm performance. Secondary outcomes: the proportion of participants achieving valid algorithm-based prediction, EMA questionnaire adherence, risk-alert opening rate, frequency of intervention library access, percentage weight loss, frequency of dietary lapses, and perceived effectiveness ratings of risk alerts. Measurement methods: Algorithm performance was evaluated by comparing the algorithm’s predictions with dietary lapse and non-lapse events reported in participants’ EMA questionnaires, with performance indices such as true positives and false negatives calculated and analyzed using the chi-square test. Body weight was remotely and automatically collected using the Yunmai Bluetooth-enabled smart scale provided by the research team, and percentage weight loss from baseline to mid-intervention and post-intervention was calculated. Dietary lapse frequency and EMA questionnaire adherence were automatically recorded and summarized by the OnTrack application. Risk-alert opening rate and frequency of intervention library access were automatically recorded and computed by the OnTrack system. Perceived effectiveness of risk alerts was assessed using ratings collected through the end-of-day questionnaire. | Intergroup differences:  Algorithm performance: The OT-L group had a higher number of true positive predictions and a lower number of false negatives, with a superior sensitivity (77.7%) compared with the OT-S group (71.6%); the OT-S group had a slightly higher specificity (84.4%). The difference in algorithm performance between the two groups was statistically significant (χ²(3)=120.29, p<0.001), while the overall accuracy was comparable between the two groups (79.9% vs 79.7%).  EMA adherence: The OT-S group (65.4%) had a higher adherence rate than the OT-L group (60.5%). The non-inferiority test failed to meet the standard, with a statistically significant difference observed between the groups.  Other indicators: No statistically significant differences were found between the two groups in terms of risk alert open rate, intervention library access frequency, perceived effectiveness of risk alerts, percentage of weight loss, and frequency of dietary lapses. Intragroup differences:  Common changes: Both groups achieved a significant reduction in body weight (an overall average weight loss of 3.4%), and the frequency of dietary lapses decreased significantly with the progression of the intervention. The adherence rate to EMA questionnaires and the open rate of risk alerts both showed a continuous declining trend as the study weeks advanced.  No specific intragroup differences: Neither group exhibited significant time-dependent differentiated fluctuations in indicators such as algorithm performance, application usage rate, and perceived effectiveness within the group; the improvement trends of core behavioral outcomes were consistent between the two groups. |
| Burke et al^[[30](file:///C:/Users/HP/Desktop/eeeee888.docx#_nebF57F5016_F528_4379_921D_2D2EBA34027F)]^. 2022, USA | In the SMARTER trial, self-monitoring data on dietary intake, physical activity, and body weight were synchronized to the mobile platform in real time. When tailored feedback was generated based on the most recent self-monitoring data and the participant was within their usual waking hours, the app delivered up to three feedback notifications per day via smartphone push messages; messages were automatically withdrawn if not opened within 1 hour. In parallel, the study backend monitored for two consecutive weeks without valid self-monitoring data, which supported the triggering of staff-initiated email outreach and engagement reminder messages. | Data sources: daily dietary self-monitoring data, daily body weight data, and daily physical activity data.  Software: the SMARTER application developed by the research team and the Fitbit app.  Hardware: participants’ own smartphones, a wrist-worn activity tracker (Fitbit Charge 2), and a smart scale. | 12 months | Primary outcome: percentage change in body weight from baseline to 12 months. Secondary outcomes: the proportion of participants achieving ≥5% weight loss, feedback-message opening rate, and the proportion of days meeting the calorie target, defined as days on which daily energy intake fell within 85%–115% of the prescribed target, provided that the participant had recorded more than 50% of the target caloric intake for that day. Measurement methods: body weight was obtained either through in-person assessment or remotely using a smart scale provided by the study. After March 2020, 12-month body weight data collection shifted from in-person measurement to remote assessment. Dietary intake was recorded via the Fitbit app. Physical activity was recorded using a Fitbit wrist-worn activity tracker. The feedback-message opening rate and related engagement metrics were automatically recorded and calculated by the SMARTER system. | Intergroup Differences:  Percentage of weight change: The intervention group (SM+FB) was -2.12%, and the control group (SM) was -2.39%, with an intergroup difference of -0.27% (95% CI: -1.57% to 1.03%, p = 0.68).  Proportion of participants with ≥5% weight loss: 26.3% in the intervention group versus 29.1% in the control group (χ² = 0.49, p = 0.49).  Absolute weight change: -1.98 kg in the intervention group versus -2.34 kg in the control group (p = 0.50).  Proportion of days meeting calorie targets: Significantly higher in the intervention group than in the control group (intergroup difference: 4.43%, 95% CI: 0.41% to 8.45%, p = 0.03).  Retention rate: 80.5% in the intervention group versus 76.5% in the control group (χ² = 1.18, p = 0.28). Intragroup Differences:  Percentage of weight change: Both groups exhibited a significant reduction in weight at 12 months compared with baseline (intervention group: -2.12%, 95% CI: -3.04% to -1.21%, p < 0.001; control group: -2.39%, 95% CI: -3.32% to -1.47%, p < 0.001).  Absolute weight change: Body weight decreased from baseline in both groups (intervention group: -1.98 kg; control group: -2.34 kg).  Proportion of days meeting calorie targets: The proportion decreased significantly over time in both groups (p < 0.001), with a smaller magnitude of decline in the intervention group (p < 0.001).  Correlation of feedback message open rate: In the intervention group, the open rate of feedback messages was significantly correlated with the degree of weight loss (b = -0.10, 95% CI: -0.13 to -0.07, p < 0.001) and adherence to calorie targets (p < 0.001). |
| Burke et al^[[31](file:///C:/Users/HP/Desktop/eeeee888.docx#_nebBBF9B20D_99F7_4852_93C3_BDCF8651FA5A)]^. 2021, USA | In the SMARTER trial, self-monitoring data on dietary intake, physical activity, and body weight were synchronized to the mobile platform in real time. When tailored feedback was generated based on these real-time monitoring data, the SMARTER app delivered three feedback notifications per day via smartphone push messages. In parallel, the study backend monitored self-monitoring lapses or periods of more than two consecutive weeks without valid monitoring data, which supported the triggering of staff-initiated email reminders. | Data collected: daily dietary records (including caloric intake, fat intake, and added sugar intake), daily body weight data, and daily physical activity data (including step count, active minutes, and sedentary time).  Software: the SMARTER app and the Fitbit app.  Hardware: smartphone, Fitbit Charge 2 wrist-worn activity tracker, and smart scale. | 6 months | Primary outcomes: percentage change in body weight and absolute change in body weight (kg) from baseline to 6 months. Secondary outcomes: the proportion of participants achieving ≥3% and ≥5% reduction in baseline body weight from baseline to 6 months; changes in BMI, body fat percentage, and waist circumference from baseline to 6 months; and changes in systolic blood pressure and diastolic blood pressure over the same period. Measurement methods: Body weight, body fat percentage, and BMI were assessed using a Tanita scale/body composition analyzer. Remote assessment: after March 17, 2020, 6-month body weight and body fat percentage data for 83 participants were collected using a smart scale. Waist circumference and blood pressure were measured only in person using standardized procedures. Dietary intake was recorded via the Fitbit app, and physical activity was automatically recorded using the Fitbit Charge 2 wrist-worn activity tracker. Intervention engagement–related metrics were automatically recorded and calculated by the SMARTER system. | Intragroup Differences：  Percentage of weight change: SM+FB: -3.16% (95% CI: -3.85% to -2.47%, p < 0.0001); SM: -3.20% (95% CI: -3.86% to -2.54%, p < 0.0001)  Absolute weight change: SM+FB: -2.95 kg (95% CI: -5.46 to -0.44, p = 0.021); SM: -3.05 kg (95% CI: -5.86 to -0.24, p = 0.034)  Waist circumference in females: SM+FB: -3.18 cm (p = 0.012); SM: -2.73 cm (p = 0.031) Intergroup Differences：  Percentage of weight change: -0.04% (95% CI: -0.99% to 0.91%, p = 0.940)  Absolute weight change: 0.04 kg (95% CI: -0.99 to 0.91, p = 0.869)  Proportion of participants with ≥5% weight loss: SM+FB: 31.9%, SM: 28.3% (χ², p = 0.381)  Proportion of participants with ≥3% weight loss: 44.6% in both groups (χ², p = 0.999)  All indicators including waist circumference, BMI, body fat percentage and blood pressure: p-values were all > 0.05 between the two groups |
| Beasley et al^[[29](file:///C:/Users/HP/Desktop/eeeee888.docx#_neb5907E13E_89E3_4716_8E51_D575021407C7)]^. 2009, USA | The DietMatePro PDA application (installed on the Palm Zire 21 device) was used to monitor dietary intake data in real time, including food type, portion size, and nutrient composition. When a participant reached a preset mealtime but had not yet entered dietary intake for that eating occasion, the system immediately delivered an auditory prompt reminding the user to record their food intake. In parallel, the PDA backend continuously tracked the discrepancy between recorded dietary intake and individualized targets (i.e., energy, total fat, saturated fat, and cholesterol), thereby supporting the delivery of tailored feedback, including comparisons between actual intake and prescribed goals and adherence-related prompts. The system also allowed users to save frequently consumed meal templates and to manually enter unlisted foods and nutrition label information. | Data collected: multidimensional dietary behavior monitoring data, including food type and portion size; 24-hour dietary recall data for comparison; anthropometric measurements including body weight and waist circumference; and intervention- and usage-related data, such as recording frequency and protocol adherence.  Software: DietMatePro application.  Hardware: Palm Zire 21 personal digital assistant (PDA) and a Detecto physician beam scale located at the research center. | 4 weeks | Primary outcomes: Agreement between dietary records and 24-hour dietary recalls with respect to energy intake and individual nutrient intakes; Adherence to the Ornish low-fat dietary regimen. Secondary outcomes: Change in body weight (lb); Change in waist circumference (inches); Completeness of dietary records, expressed as the proportion of recorded days with plausible energy intake; Participants’ subjective ratings of the dietary recording method; Changes in energy and nutrient intake from baseline to post-intervention. Measurement methods: Agreement in dietary recording was evaluated by comparing energy and nutrient intake data obtained from DietMatePro/PDA or paper-based food records with those from concurrent 24-hour dietary recalls. Dietary regimen adherence was assessed as the proportion of days during week 4 on which participants simultaneously met the targets for energy intake, total fat (<15% of total energy), saturated fat (<7% of total energy), and cholesterol (<200 mg). Anthropometric measures: body weight was measured using a Detecto physician beam scale, and waist circumference was measured using a tension-controlled tape measure. Completeness of dietary records was assessed as the proportion of recorded days with energy intake within a plausible range. Subjective evaluation was assessed using a 5-point Likert scale. Changes in nutrient intake were derived from 24-hour dietary recalls collected in weeks 1 and 4; energy and nutrient data were analyzed using ESHA Food Processor SQL version 9.1.2, and baseline-to-post-intervention differences were calculated. | Between-group differences:  Dietary adherence: participants in the PDA group met the dietary targets on 43% of days, compared with 28% of days in the paper diary group (P = 0.039). The PDA group also showed significantly greater reductions in energy intake and fat intake (P < 0.05). Recording validity: correlations between recorded intake data and 24-hour dietary recalls were higher in the paper diary group (0.63–0.83) than in the PDA group (0.41–0.71) (P < 0.05). Anthropometric outcomes: waist circumference decreased by 1.0 inch in the PDA group and by 0.5 inch in the paper diary group (P = 0.04). Although weight loss was greater in the PDA group, the between-group difference was not statistically significant. Record completeness: during week 1, the proportion of missing or implausible data was higher in the PDA group (20%) than in the paper diary group (8%) (P = 0.03); however, no significant between-group difference was observed over the full study period.  Within-group differences:  PDA group: from baseline to week 4, energy intake and nutrient intake decreased significantly, and both body weight and waist circumference were significantly reduced (P < 0.05); recording proficiency also improved over time. Paper diary group: from baseline to week 4, energy intake and fat intake also decreased significantly, although the magnitude of reduction was smaller. Body weight and waist circumference decreased only slightly and did not reach statistical significance, while recording quality showed a slight decline. |
| Haapala et al^[[28](file:///C:/Users/HP/Desktop/eeeee888.docx#_neb2C6756B1_FACB_4A87_A65C_B22BCC20C895)]^. 2009, Finland | The Weight Balance mobile program continuously monitored participants’ daily self-reported body weight and progress toward target weight goals. When a participant submitted their body weight for the day via text message or the website and the reported value was consistent with the expected target-weight trajectory, the system immediately delivered a personalized text-message feedback to the mobile phone. In parallel, the program backend continuously tracked the frequency of user engagement with the program (e.g., number of weekly weight reports) and deviations from expected weight-loss progress, thereby informing adjustments to the feedback content, such as short-term goal reminders. The program also allowed users to log dietary intake via the website, view visualized weight-loss progress charts, and access links to health, nutrition, and physical activity information. | Data collected: anthropometric measurements such as body weight and waist circumference; daily self-reported body weight data; multidimensional weight-management behavioral data, including dietary intake, physical activity, and dieting self-efficacy; and program engagement data, such as frequency of use and user satisfaction.  Software: Weight Balance app and Nutrica® 3.1. Hardware: mobile phone. | 12 months | Primary outcomes: Change in body weight (kg); Change in waist circumference (cm). Secondary outcomes: changes in dieting self-efficacy scores; changes in frequency scores for consumption of energy-dense foods; program use/contact frequency; participants’ satisfaction ratings regarding the usability and practical utility of the program; and changes in the frequency of leisure-time physical activity. Measurement methods: Anthropometric measures: assessed through in-person measurements. Dieting self-efficacy: evaluated using a 10-item scale. Consumption of energy-dense foods: assessed using a questionnaire covering the frequency of intake across eight categories of energy-dense foods. Program use/contact frequency: assessed by self-report, including the frequency of weight reporting and website use, and quantified according to predefined scoring criteria (e.g., daily weight reporting counted as 7 times per week). Satisfaction: assessed using the Finnish school grading scale (4–10). Physical activity: assessed using the Finnish National Health Survey questionnaire. | Between-group differences: Body weight: the intervention group lost 4.5 kg, whereas the control group lost only 1.1 kg (P = 0.006). The proportions of participants achieving clinically meaningful weight loss (≥5% and ≥10%) were also markedly higher in the intervention group than in the control group. Waist circumference: the intervention group showed a reduction of 6.3 cm, compared with 2.4 cm in the control group (P = 0.0001). Dietary behavior: intake of energy-dense foods decreased significantly in the intervention group, whereas no change was observed in the control group (P = 0.03). Participants in the intervention group also demonstrated more active health-information–seeking behavior.  Within-group differences: Intervention group: from baseline to 3, 6, 9, and 12 months, both body weight and waist circumference decreased highly significantly (P < 0.0001). The frequency of physical activity increased, whereas dieting self-efficacy showed a slight decline (P = 0.05). Control group: from baseline to 12 months, there were no statistically significant changes in body weight, waist circumference, or dietary/behavioral indicators; only a slight decline in self-efficacy was observed. |
| Spanakis et al^[[43](file:///C:/Users/HP/Desktop/eeeee888.docx#_nebBECB419B_0740_4AE5_AEB4_34B70CAF4482)]^. 2017, Netherlands | The Think Slim mobile application used ecological momentary assessment (EMA) to collect real-time contextual data, including affect, food cravings, location, and activity. When a participant completed a random-sampling questionnaire or a pre-eating event-based questionnaire and the reported data matched predefined predictive rules for unhealthy eating (e.g., evening + at home + craving for unhealthy food), the system immediately delivered an adaptive feedback notification via smartphone, including a warning about unhealthy eating risk and tailored behavioral suggestions. In parallel, the app backend continuously monitored the frequency of rule activation and participants’ responses to feedback, thereby supporting the refinement of the personalized rule library and the optimization of rule matching within the assigned eating-behavior subgroup. New users were assigned to one of six predefined subgroups after a 1-week monitoring period, and subsequent feedback was generated based on both subgroup-level rules and the user’s person-specific salient rules. | Data collected: multidimensional diet-related behavioral monitoring variables. Random-sampling assessments were conducted across eight time windows per day, each lasting approximately 2 hours. Software: the Think Slim application. Hardware: iPhone. | Study duration: 2 weeks in Study I and 8 weeks in Study II. | Primary outcome: Unhealthy eating events.  Secondary outcomes/process evaluation measures: Rule-triggering frequency; Eating behavior profiles.  Measurement method: All data were collected using the Think Slim mobile application in accordance with the principles of ecological momentary assessment (EMA). | Within-profile differences: Participants within each of the six eating behavior profiles showed a high degree of within-group homogeneity in their eating behavior characteristics. After inclusion of new participants from Study II, the increases in mean within-cluster distance were 1.13% for Profile 2, 2.91% for Profile 1, 2.66% for Profile 4, 2.27% for Profile 5, 3.94% for Profile 3, and 4.61% for Profile 6. Between-profile differences: The proportion of unhealthy-eating rules varied across profiles, at 19.0% for Profile 2, 27.8% for Profile 3, 39.6% for Profile 5, 42.0% for Profile 1, 52.4% for Profile 6, and 55.6% for Profile 4. The mean daily frequency of warning-rule activation was 0.42 in Profile 2, 0.83 in Profile 3, 0.89 in Profile 1, 1.21 in Profile 5, 1.27 in Profile 4, and 1.82 in Profile 6. The proportion of overall rule activation was 5.30% for Profile 2, 10.40% for Profile 3, 11.10% for Profile 1, 15.10% for Profile 5, 15.90% for Profile 4, and 22.80% for Profile 6. The number of active rules also differed by profile, with 8 rules in Profile 4, 10 in Profile 2, 13 in Profile 5, 14 in Profiles 1 and 6, and 15 in Profile 3. In Study II, the proportion of unhealthy-rule activation was 3.05% for Profile 2, 8.31% for Profile 3, 9.63% for Profile 4, 11.84% for Profile 1, 15.02% for Profile 5, and 16.75% for Profile 6. Some rules overlapped across profiles. |
| Hermsen et al^[[35](file:///C:/Users/HP/Desktop/eeeee888.docx#_neb48E91B0C_6BD9_40EE_9F2F_D8F72D448E41)]^. 2019， Netherlands | The smart fork continuously monitored the time interval between two consecutive eating actions (bite rate) using an integrated capacitive sensor. If the system detected that the interval between two bites was shorter than 10 seconds, indicating an excessively rapid eating pace, the fork handle immediately delivered vibrotactile feedback accompanied by an LED light signal. This form of physical feedback prompted participants to adjust their eating rhythm and prolong chewing time until their eating rate returned to the preset healthy threshold. | Data collected: bite rate, success ratio, and body weight.  Software: a dedicated secure online web portal/dashboard.  Hardware: the 10sFork. | 4 weeks | Primary outcomes: bite rate, success ratio, and body weight.  Secondary outcomes: meal duration, total number of bites per meal, and inter-bite interval.  Measurement methods: bite rate, success ratio, meal duration, total number of bites per meal, and inter-bite interval were all automatically recorded by the 10sFork smart fork and calculated via the SlowConnect server. Body weight was measured by participants’ dietitians at their respective practice sites using standardized equipment at baseline (T1), post-intervention at 4 weeks (T2), and follow-up at 8 weeks (T3). Weight change was presented in terms of body mass index (BMI). | Between-group differences: Bite rate: At T2, both the VFC group (6.2 → 4.4 bites/min) and the VFC+ group (5.9 → 4.1 bites/min) showed significantly lower bite rates than the NFC group (6.4 → 5.6 bites/min) (F(4,1886) = 3.49, p < 0.01). No significant difference was observed between the VFC and VFC+ groups. Success ratio: At T2, both the VFC group (43.2% → 65.3%) and the VFC+ group (45.4% → 68.2%) had significantly higher success ratios than the NFC group (45.2% → 44.3%) (F(4,1887) = 21.05, p < 0.0001). No significant difference was found between the VFC and VFC+ groups. BMI: At T2, both the VFC group (31.1 → 30.6 kg/m²) and the VFC+ group (31.6 → 31.2 kg/m²) had significantly lower BMI values than the NFC group (31.7 → 31.9 kg/m²) (F(4,213) = 3.00, p = 0.02). No significant difference was observed between the VFC and VFC+ groups. Inter-bite interval: At T2, the VFC group (13.1 → 19.3 s) and the VFC+ group (14.3 → 21.3 s) both showed significantly longer inter-bite intervals than the NFC group (13.1 → 14.7 s) (F(4,1913) = 3.75, p < 0.01). Within-group differences: VFC group: Bite rate: T1 6.2 → T2 4.4 → T3 4.9 bites/min (T1 vs T2/T3, p < 0.0001) Success ratio: T1 43.2% → T2 65.3% → T3 55.3% (T1 vs T2/T3, p < 0.0001) BMI: T1 31.1 → T2 30.6 → T3 30.3 kg/m² (T1 vs T2, p < 0.001; T1 vs T3, p < 0.0001) VFC+ group: Bite rate: T1 5.9 → T2 4.1 → T3 4.6 bites/min (T1 vs T2/T3, p < 0.0001) Success ratio: T1 45.4% → T2 68.2% → T3 57.5% (T1 vs T2/T3, p < 0.0001) BMI: T1 31.6 → T2 31.2 → T3 30.9 kg/m² (T1 vs T2, p < 0.02; T1 vs T3, p < 0.001) NFC group: Bite rate: T1 6.4 → T2 5.6 bites/min (p < 0.05), then T3 6.2 bites/min (p = 0.33, not significantly different from baseline) Success ratio: no significant difference from baseline at any time point (p > 0.05) BMI: no significant difference between T2 and baseline (p = 0.18), whereas T3 was significantly lower than baseline (p < 0.05) |
| Valle et al^[[42](file:///C:/Users/HP/Desktop/eeeee888.docx#_nebFDF6038E_AED5_434B_A5CB_E62D45311BBC)]^. 2025, USA | At four daily decision points (7:00, 10:00–12:00, 14:00–16:00, and 19:00–21:00), the system first assessed whether the participant had failed to meet the relevant behavioral target. If the participant had not weighed themselves, a weight-weighing reminder was triggered. If the participant’s minutes of physical activity had not reached 50% of the daily goal, an activity prompt was delivered. If the participant’s intake of red-category foods had reached 80% of the daily limit, a red-food restriction reminder was triggered. At each decision point, the system then randomly delivered one type of behavior change technique (BCT) message, such as a social comparison message (e.g., “Yesterday, 55% of participants met the target—you can do it too!”). | Data collected: body weight (via the Fitbit Aria smart scale), minutes of physical activity (via the Fitbit Alta activity tracker), red-food intake (logged in the Nudge app), and goal progress.  Software: the Nudge app (iOS only) and the Fitbit API.  Hardware: iPhone, Fitbit activity tracker, and wireless smart scale. | 12 weeks | Primary outcome: daily attainment of weight-related behavioral goals. Secondary outcomes: completion of red-food tracking, activity-tracker wear adherence, daily active minutes, total daily red-food intake, receipt of intervention messages, message viewing, 12-week weight change, and percentage weight loss at 12 weeks.  Measurement methods: Daily goal attainment: Weighing behavior: assessed using the Fitbit smart scale. Active-minutes goal attainment: assessed using data recorded by the Fitbit activity tracker. Red-food restriction: assessed through food logs entered in the Nudge app. Completion of red-food tracking: defined as complete when breakfast, lunch, and dinner were all logged or when the red-food limit had already been reached. Tracker wear adherence: automatically determined based on Fitbit-derived data. Active minutes and total red-food intake: extracted as continuous measures from the Fitbit tracker and Nudge app logs, respectively. Intervention-related measures: Message receipt: automatically recorded by the system at the four daily decision points. Message viewing: determined based on app interaction data. Weight-loss outcomes: Body weight was measured using a calibrated scale. | Receipt of any message vs no message received: only red-food tracking volume was reduced (OR = 0.96, 95% CI: 0.94–0.98).  Message viewed vs not viewed: viewing a message was associated with higher odds of meeting the weighing target (OR = 1.91), the active-minutes target (OR = 1.63), and the red-food restriction target (OR = 2.09), with all comparisons reaching statistical significance (P < .05).  Weight change at 12 weeks: the mean change in body weight was −2.7 (SD 3.6) kg. The total number of goals achieved was significantly associated with greater weight loss (B = −0.05, P < .0001). |
| Dorsch et al^[[32](file:///C:/Users/HP/Desktop/eeeee888.docx#_neb9CEC5D77_1796_41F8_B5A1_3C6D4A749FE1)]^. 2020, USA | Using geofencing technology, the system delivered context-specific prompts when participants entered a grocery store, restaurant, or home environment, as identified by GPS-based matching with a point-of-interest (POI) database. In the grocery store setting, participants received suggestions for lower-sodium alternatives to commonly consumed high-sodium foods; in the restaurant setting, they received prompts highlighting lower-sodium menu options; and in the home setting, they received low-sodium cooking tips. In addition, if the app-recorded sodium intake exceeded 2000 mg during the previous 24 hours, a sodium-intake control reminder was triggered on the following day. | Data collected: geolocation data (GPS), food sodium content, and user-specific alternatives to frequently consumed high-sodium foods.  Software: the LowSalt4Life app (iOS only) and the Nutritionix API (nutrition database).  Hardware: iPhone and a universal product code (UPC) scanner. | 8 weeks | Primary outcome: change in 24-hour urinary sodium excretion. Secondary outcomes: Sodium intake–related outcomes: change in 24-hour urinary sodium excretion, change in dietary sodium intake, and change in sodium screener scores; Change in blood pressure; Self-efficacy; Number of app push notifications received; Use of nutrition-information search functions (including autocomplete searches and barcode-scanning frequency).  Measurement methods: Estimated 24-hour urinary sodium excretion: assessed using fasting first-morning urine samples collected at baseline and 8 weeks. 24-hour urinary sodium excretion: measured using 24-hour urine collections obtained at baseline and 8 weeks. Estimated sodium intake: assessed at baseline and 8 weeks using a food frequency questionnaire (FFQ), ASA24, and a sodium screener. Blood pressure: measured through home self-monitoring. Self-efficacy: assessed using the SCFLDS scale. App use data: automatically recorded through the Nutritionix API. | Using the Kawasaki equation to estimate 24-hour urinary sodium excretion, the intervention group showed a reduction of 462 mg, whereas the control group showed an increase of 381 mg (P = 0.03). Based on FFQ-assessed sodium intake, the intervention group showed a reduction of 1553 mg, compared with a reduction of 515 mg in the control group (P = 0.01). For measured 24-hour urinary sodium excretion, the intervention group showed a reduction of 637 mg, whereas the control group showed a reduction of 322mg; however, this between-group difference was not statistically significant (P = 0.47). For systolic blood pressure, the intervention group decreased by 7.5 mmHg, compared with a decrease of 0.7 mmHg in the control group; this difference was also not statistically significant (P = 0.12). |
| Dorsch et al^[[36](file:///C:/Users/HP/Desktop/eeeee888.docx#_neb2D368451_5A6D_43C0_B61F_A5B2558A5ACD)]^，2025 , USA | At four participant-selected daily time points (e.g., 7:00, 12:00, 18:00, and 21:00), notifications were delivered with a 25% randomization probability. If the Fitbit smartwatch detected fewer than 15 steps in the previous hour, indicating sedentary behavior, the system triggered a prompt encouraging the participant to get up and walk for 10 minutes. If the participant had preset the following 24 hours as a grocery-shopping period, the system delivered a low-sodium supermarket shopping list. In addition, when real-time weather conditions indicated a temperature of >25°C with no precipitation, the system preferentially delivered outdoor physical activity suggestions. | Data collected: blood pressure, daily step count, sodium intake, weather conditions, time of day, and mobility-related data. Software: the myBPmyLife app (embedded within the MyDataHelps platform), the Fitbit app, and the Omron blood pressure monitoring app. Hardware: a Fitbit Versa 2 smartwatch, an Omron Evolv BP7000 Bluetooth-enabled blood pressure monitor, and a smartphone. | 6 months | Primary outcome: change in systolic blood pressure. Secondary outcomes: change in diastolic blood pressure, change in mean daily step count, change in daily dietary sodium intake, cumulative score for antihypertensive medication adjustments, and, in the intervention group, app engagement/use and system usability. Measurement methods: Blood pressure was measured using a Bluetooth-enabled blood pressure monitor (Omron Evolv BP7000). Daily step count was recorded using a Fitbit Versa 2 smartwatch. Dietary sodium intake was assessed using the Block Sodium Screener. Medication adjustment score was calculated by recording changes in antihypertensive medications from baseline to 6 months, including newly added medications (+2), dose increases (+1), medication discontinuation (−1), and dose reductions (−1), and then summing these values to derive a cumulative score. App-related outcomes in the intervention group were automatically recorded through the MyDataHelps app, and system usability was assessed at the end of the study using the System Usability Scale (SUS). | Between-group differences: there was no significant difference in the change in systolic blood pressure (SBP) between the intervention and control groups (−5.2 mmHg vs −5.7 mmHg, p = 0.76). However, daily step count increased by 170 steps in the intervention group, whereas it decreased by 319 steps in the control group (p = 0.040). In addition, dietary sodium intake decreased by 1145 mg in the intervention group compared with 860 mg in the control group (p = 0.002).  Within-group differences: both groups showed significant reductions in SBP and diastolic blood pressure (DBP) (p < 0.05). Improvements in behavioral outcomes, particularly step count and sodium intake, were more pronounced in the intervention group. |
| Conroy et al^[[41](file:///C:/Users/HP/Desktop/eeeee888.docx#_nebA8CEC2EB_8D81_4C71_A6BB_D4A5471A9046)]^，2020, USA | The H2OPal smart water bottle assessed fluid intake every 30 minutes. If less than 150 mL of water had been consumed within the preceding 30-minute interval and the participant was not within a designated do-not-disturb period, the system triggered a notification via the mobile app. In parallel, the Fitbit smartwatch monitored whether there had been no hand movements associated with drinking behavior for 2 consecutive hours, which further supported the triggering of a reminder prompt. | Data collected: fluid intake volume. Software: the sipIT app, H2OPal app, Fitbit app, and the Fitabase backend platform. Hardware: the H2OPal smart water bottle, Fitbit Versa smartwatch, and iPhone. | 3 weeks | Primary outcomes: automaticity of fluid intake behavior and the occurrence of fluid intake–related barriers. Secondary outcomes: intervention acceptability, intervention use–related indicators, and user experience feedback. Measurement methods: Automaticity of fluid intake behavior was assessed at baseline, 1 month, and 3 months using a 4-item scale. Occurrence of barriers was assessed at baseline and during monthly follow-up from months 1 to 3 using a barrier checklist. Acceptability was evaluated based on retention rates at 1 month (90%) and 3 months (87%); system usability was assessed at 1 month using the System Usability Scale (SUS), and satisfaction was measured using four custom-designed items. Intervention use was automatically recorded through the sipIT semi-automated monitoring system—integrating the H2OPal smart water bottle, Fitbit smartwatch gesture detection, and mobile app–based self-monitoring—including the number of drinking events, the number of notifications delivered, and the median number of daily notifications. Participants also provided rating-scale reports on perceived improvements in fluid intake and their success in meeting the recommended intake target. User experience was explored through semi-structured interviews. | Within-group changes: automaticity of fluid intake behavior increased significantly from baseline to 1 month (Cohen’s d = 0.50, p < 0.05) and from baseline to 3 months (Cohen’s d = 0.64, p < 0.01). The barrier of “lack of thirst” also decreased, from 51.6% at baseline to 23.1% at 1 month (χ² = 4.86, p < 0.05). Acceptability: at 1 month, the mean system usability score was 4.4 ±0.7, and 96% of participants reported that they had been successful in increasing their fluid intake. |
| Juarascio et al^[[40](file:///C:/Users/HP/Desktop/eeeee888.docx#_neb8A8E9441_404E_4E58_B2E7_3F9DC2DF417D)]^， 2021, USA | The app monitored participants’ self-reported data in real time. When a participant reported a negative mood rating of 4/5 and had not used an emotion-regulation strategy, the system triggered an emotion-regulation skills prompt (e.g., “Try using an emotion diary to record your feelings.”). If the participant had not eaten for 4 consecutive hours, indicating that the regular eating target had not been met, the system delivered a regular-eating intervention prompt (e.g., “Identify the reason for not eating and make a plan for your next two meals.”). In addition, if no coping behavior was recorded within 30 minutes after reporting an urge to binge eat, the system triggered a follow-up skills-use reminder. | Data collected: eating events (including type, timing, and food consumed), affective state, eating disorder behaviors/urges, and skills use. Software: the CBT+ smartphone application (patient-facing app) and the CBT+ clinician portal (clinician-facing platform). Hardware: iPhone. | 16 weeks | Primary outcomes: improvement in the acquisition and application of cognitive behavioral therapy (CBT) skills and reduction in bulimia nervosa (BN) symptom severity. Secondary outcomes: intervention feasibility, intervention acceptability, and changes in quality of life. Measurement methods: CBT skill acquisition and use were assessed using electronic self-monitoring forms completed via the CBT+ app, in conjunction with relevant item scores from the Restraint subscale of the Eating Disorder Examination (EDE) and the Difficulties in Emotion Regulation Scale (DERS). Improvement in BN symptoms was assessed using the Eating Disorder Examination (EDE). Intervention feasibility was evaluated using app-generated usage data automatically recorded by the CBT+ app. Intervention acceptability was assessed using a 6-point Likert scale, supplemented by qualitative feedback collected through telephone interviews and weekly scheduled questionnaires. Quality of life was assessed using a quality-of-life scale. | Feasibility: participants completed a mean of 3.13 ± 1.03 self-monitoring entries per day, and the app was used on 86.1% of study days. The incidence of technical problems was low, with only 5% of participants reporting such issues. Acceptability: the median rating of intervention usefulness was 5/6 among both patients and clinicians. Qualitative feedback indicated that the intervention was valued for enhancing accountability and providing timely skills reminders, although some participants noted that certain content was repetitive. Clinical outcomes: among treatment completers, binge-eating episodes and compensatory behaviors decreased significantly, and the global Eating Disorder Examination (EDE) score declined to within 1 standard deviation of community norms. |
| Juarascio et al^[[37](file:///C:/Users/HP/Desktop/eeeee888.docx#_neb5DFEB74E_FEDD_45A4_9E42_D49DDCA36C89)]^， 2023, USA | The CBT+ smartphone application continuously collected self-monitoring data in real time. When the monitoring records indicated restrictive eating or an urge to engage in binge eating or compensatory behaviors, and the participant had completed data entry, the app delivered an in-app just-in-time intervention. In parallel, clinicians manually configured individualized push-notification rules based on each participant’s symptom profile; when a customized high-risk time window was reached or when more than 5 hours had elapsed without completion of a self-monitoring entry, the system supported the delivery of personalized smartphone push notifications. | Data collected: self-monitoring data across four domains, including eating behaviors, binge-eating and compensatory symptoms, use of CBT treatment skills, and momentary affective states. Software: the smartphone-based self-monitoring application and the CBT-E treatment support platform. Hardware: smartphone. | 16 weeks | Primary outcomes: frequency of binge-eating episodes, frequency of compensatory behaviors, and overall severity of eating disorder psychopathology. Measurement methods: the Eating Disorder Examination (EDE) and semi-structured clinical interviews. Secondary outcomes: use of CBT treatment skills, feasibility of the CBT+ system, and acceptability of the CBT+ system. Measurement methods: weekly self-reported ratings of skill use; app backend–derived usage data; and post-treatment assessment using the Technology Acceptance Model (TAM) questionnaire in combination with semi-structured qualitative interviews. | Within-group changes: Both groups showed significant improvement over time. The frequency of binge-eating and compensatory behaviors, as well as all EDE subscale scores and the global EDE score, decreased significantly from baseline. The use of CBT skills—including regular eating and emotion regulation strategies—increased significantly from the early phase of treatment to week 16, and these improvements were maintained at the 3-month follow-up. Between-group differences: There were no statistically significant between-group differences. The JITAIs-On and JITAIs-Off groups did not differ significantly in improvements in binge-eating and compensatory behaviors, changes in EDE scores, or increases in CBT skill use. The only significant between-group difference was that the JITAIs-On group reported significantly higher perceived ease of use of the CBT+ app than the control group, with a medium effect size. During follow-up, the JITAIs-Off group showed a 76% rebound in mean binge-eating frequency, whereas no rebound was observed in the JITAIs-On group; however, this trend did not reach statistical significance. |
| Nechita & Matu^[[47](file:///C:/Users/HP/Desktop/eeeee888.docx#_neb30D91FC2_0F8D_4D53_8ECE_327704C41C0B)]^, 2026, Romania | The Expiwell smartphone app assessed affective states and eating-related behaviors five times per day through semi-random prompts delivered between 8:30 AM and 10:30 PM. When a participant reported a score of ≥3 (moderate to severe) on general shame, body-related shame, or eating-related shame, and responded within the active response window (within 20 minutes of prompt delivery), the app immediately delivered an in-app self-compassion intervention prompt, accompanied by a dedicated text-entry field. In parallel, the app backend monitored nonresponse to prompted questionnaires within the 20-minute window as an indicator of low adherence, thereby supporting the delivery of stage-specific in-app adherence feedback. | Data collected: shame intensity ratings, mood/affective states, and records of eating behaviors. Software: a dedicated mobile intervention application (Expiwell). Hardware: smartphone. | 1 WEEKS | Primary outcomes: binge-eating behavior and body-checking behavior. Secondary outcomes: dietary restraint, compensatory behaviors, excessive exercise, eating self-efficacy, general shame, body shame, eating-related shame (three dimensions), and negative affective states such as fear, hostility, tension, distress, and guilt. Measurement methods: ecological momentary assessment (EMA) was used, with real-time self-reports collected on a 5-point Likert scale. Assessments were administered five times per day using a semi-random sampling schedule over a 1-week period, with all data collected through the Expiwell mobile application. | Between-group differences: Binge-eating behavior: after high-shame episodes, binge-eating increased in the control group but remained stable in the intervention group (b = -0.657, t = -2.603, p = 0.009). Estimated means: control 2.17 (SE = 0.11) vs intervention 1.89 (SE = 0.13). Body-checking behavior: the control group had a higher baseline level (b = -0.526, t = -2.368, p = 0.020). After high-shame episodes, body-checking increased in the control group but not in the intervention group; the interaction was significant (b = -0.784, t = -3.187, p = 0.001). Estimated means: control 3.12 (SE = 0.15) vs intervention 2.41 (SE = 0.17). High-shame episodes: the intervention group had a lower likelihood on day 6 (OR = 0.17, 95% CI 0.04–0.85, p < 0.05) and day 7 (OR = 0.16, 95% CI 0.03–0.99, p < 0.05). Dietary restraint: no significant group effect (p > 0.05). Compensatory behaviors: no significant group effect (p > 0.05). Excessive exercise: no significant group effect (p > 0.05). Eating self-efficacy: no significant group effect (b = -0.340, t = -1.174, p = 0.240). Within-group differences: Binge-eating behavior: in the control group, binge-eating increased after high-shame episodes (1.51 ± 0.20 to 2.17 ± 0.11, p = 0.006, d = 0.53). It also increased within the day (b = 0.051, t = 4.529, p < 0.001) and decreased across the study week (b = -0.059, t = -2.690, p = 0.007). No pre–post change was found in the intervention group (1.89 ± 0.15 to 1.89 ± 0.13, p = 1.000). Body-checking behavior: in the control group, body-checking increased after high-shame episodes (2.40 ± 0.23 to 3.12 ± 0.15, p = 0.001, d = 0.54). No significant change was found in the intervention group. High-shame episodes: in the intervention group, frequency was lower on days 6 and 7 than earlier in the study (both p < 0.05); no significant time trend was found in the control group (p > 0.05). Compensatory behaviors: increased over the day in both groups (b = 0.011, t = 2.164, p = 0.031), with no pre–post event differences. Excessive exercise: increased over the day in both groups (b = 0.019, t = 2.801, p = 0.005), with no pre–post event differences. Dietary restraint: no significant changes (p > 0.05). Eating self-efficacy: no significant changes (p > 0.05). |
| Everett et al^[[39](file:///C:/Users/HP/Desktop/eeeee888.docx#_nebBC3B9BD4_7D43_4DBC_AA2C_69A3421CAE85)]^. 2018, USA | The Sweetch mobile application, in conjunction with a Bluetooth digital body weight scale (DBWS), continuously monitored physical activity (PA), body weight, and user contextual data (e.g., calendar information and location). When the machine-learning algorithm identified a contextually opportune moment for intervention—such as when the user’s calendar indicated free time, when the user was located in a park or another activity-conducive setting, or when the user was at home—and the user had not yet achieved the interim PA or weight-management goal, the system immediately delivered a personalized push notification via smartphone, including brief activity suggestions and weight-management prompts. In parallel, the app backend continuously tracked the user’s response to notifications (i.e., whether the suggested action was carried out), thereby informing ongoing optimization of the timing, type, and content of subsequent notifications. All notifications were delivered fully automatically, without human intervention. | Data collected/outcomes: an increase in weekly physical activity of 2.8 MET-h, a mean weight loss of 1.6 kg, a median change in glycated hemoglobin (HbA1c) of −0.1%, and a System Usability Scale (SUS) score of 78%. Software: the Sweetch application (an AI-driven fully automated platform). Hardware: a digital body weight scale (DBWS) and a smartphone. | 3 months | Primary outcomes: physical activity level, body weight, body mass index (BMI), glycated hemoglobin (HbA₁c), and fasting blood glucose. Secondary outcomes: waist circumference, blood pressure, acceptability of the platform and hardware, study retention rate, and adverse events. Measurement methods: Physical activity level was automatically captured through smartphone sensors, expressed as MET-hours per week (MET-h/week), and calculated as the difference between the mean values during the first and last 2 weeks of the study. Body weight, BMI, waist circumference, and blood pressure were measured using standardized procedures at clinical sites, and changes were calculated between baseline and the 3-month endpoint. HbA₁c and fasting blood glucose were assessed using serum samples analyzed in a central laboratory, with baseline and endpoint values obtained to determine change over time. Platform and hardware acceptability were assessed at the study endpoint using the System Usability Scale (SUS) for the app and a dedicated questionnaire for the digital scale, with scores standardized to a 0–100 scale. Study retention rate was defined as the proportion of enrolled participants who completed the study protocol. Adverse events were collected through participant self-report and end-of-study face-to-face interviews, with investigators determining their relatedness to the intervention. | Within-group changes: Body weight: at the 3-month follow-up, body weight (89.6 ± 20.8 kg) was significantly lower than at baseline (91.8 ± 20.5 kg), with a mean difference of −2.2 kg (p < 0.001). BMI: at the 3-month follow-up, BMI (32.1 ± 6.4 kg/m²) was significantly lower than at baseline (33.0 ± 6.5 kg/m²), with a mean difference of −0.9 kg/m² (p < 0.001). Waist circumference: at the 3-month follow-up, waist circumference (105.0 ± 16.6 cm) was significantly lower than at baseline (107.7 ± 16.7 cm), with a mean difference of −2.7 cm (p = 0.001). Physical activity level: at the 3-month follow-up, physical activity (15.9 ± 7.9 MET-hours/week) was significantly higher than at baseline (12.5 ± 9.4 MET-hours/week), with a mean difference of +3.4 MET-hours/week (p < 0.001). HbA1c: compared with baseline (6.0 ± 0.3%), HbA1c at the 3-month follow-up (5.9 ± 0.3%) showed a mean difference of −0.1%, which was not statistically significant (p = 0.09). Fasting blood glucose: compared with baseline (103.7 ± 7.5 mg/dL), fasting blood glucose at the 3-month follow-up (103.3 ± 6.7 mg/dL) showed a mean difference of −0.4 mg/dL, with no statistically significant difference (p > 0.05). |

EMI：Ecological momentary intervention

FVI：Fruit and vegetable intervention

AIM：Automatic Ingestion Monitor

BTS:Beyond the Scale

OT: OnTrack

WW:Weight Watchers

FS:Freestyle

BMI:Body Mass Index

OT-L: OnTrack-long

OT-S: OnTrack-short

SM:self-monitoring

SM+FB:self-monitoring plus feedback

PDA:Personal digital assistant

NFC：No Feedback Condition

VFC：Vibrotactile Feedback Condition

VFC+：Vibrotactile + Visual Feedback Condition

FFQ:Food Frequency Questionnaire

SBP:Dystolic blood pressure

DBP:Diastolic blood pressure

EDE:Eating Disorder Examination

CBT:Cognitive behavioral therapy
